# H7N7 viral infection elicits pronounced, sex-specific neuroinflammatory responses *in vitro*

**DOI:** 10.3389/fncel.2024.1444876

**Published:** 2024-08-07

**Authors:** Lea Gabele, Isabell Bochow, Nele Rieke, Christian Sieben, Kristin Michaelsen-Preusse, Shirin Hosseini, Martin Korte

**Affiliations:** ^1^Department of Cellular Neurobiology, Zoological Institute, Technische Universität Braunschweig, Braunschweig, Germany; ^2^Helmholtz Centre for Infection Research, Research Group Neuroinflammation and Neurodegeneration, Braunschweig, Germany; ^3^Helmholtz Centre for Infection Research, Nanoscale Infection Biology Group, Braunschweig, Germany; ^4^Institute of Genetics, Technische Universität Braunschweig, Braunschweig, Germany

**Keywords:** influenza A virus, microglial activation, sex, hippocampus, CNS

## Abstract

Influenza A virus (IAV) infection can increase the risk of neuroinflammation, and subsequent neurodegenerative diseases. Certain IAV strains, such as avian H7N7 subtype, possess neurotropic properties, enabling them to directly invade the brain parenchyma and infect neurons and glia cells. Host sex significantly influences the severity of IAV infections. Studies indicate that females of the reproductive age exhibit stronger innate and adaptive immune responses to IAVs compared to males. This heightened immune response correlates with increased morbidity and mortality, and potential neuronal damage in females. Understanding the sex-specific neurotropism of IAV and associated mechanisms leading to adverse neurological outcomes is essential. Our study reveals that primary hippocampal cultures from female mice show heightened interferon-β and pro-inflammatory chemokine secretion following neurotropic IAV infection. We observed sex-specific differences in microglia activation: both sexes showed a transition into a hyper-ramified state, but only male-derived microglia exhibited an increase in amoeboid-shaped cells. These disparities extended to alterations in neuronal morphology. Neurons derived from female mice displayed increased spine density within 24 h post-infection, while no significant change was observed in male cultures. This aligns with sex-specific differences in microglial synaptic pruning. Data suggest that amoeboid-shaped microglia preferentially target postsynaptic terminals, potentially reducing neuronal hyperexcitability. Conversely, hyper-ramified microglia may focus on presynaptic terminals, potentially limiting viral spread. In conclusion, our findings underscore the utility of primary hippocampal cultures, incorporating microglia, as an effective model to study sex-specific, virus-induced effects on brain-resident cells.

## 1 Introduction

Respiratory viruses, particularly influenza A viruses (IAV), impose a significant health burden, with the World Health Organization (WHO) estimating that annual IAV epidemics cause around 1 billion infections, 3–5 million cases of severe illness, and 390,000–650,000 deaths globally each year. Typically, IAV infection results in a self-limiting respiratory syndrome, but can lead to systemic complications involving the heart, the central nervous system, and other organs (Sellers et al., 2017; Joynt, 2018). Recent studies have linked IAV infection to neurodegenerative diseases such as Alzheimer’s disease (Frank-Cannon et al., 2009; Heneka et al., 2014) and Parkinson’s disease (Toovey et al., 2011; Zhou et al., 2013; Cocoros et al., 2021). Certain IAVs substrains, such as the avian subtype H7N7, are neurotropic and can invade the brain parenchyma, infecting resident cells directly. Neurological impairments from IAV may result from direct viral invasion or the excessive release of pro-inflammatory factors, leading to inflammation and tissue damage. Genetics, epigenetics, age, and sex influence the outcome of IAV infections (Morgan and Klein, 2019).

Sex-specific differences in prevalence and severity have often been overlooked in the past. Though most seasonal influenza case reports do not differentiate between sexes, evidence suggests a higher risk of severe outcomes in prepubertal males compared to age-matched females (Quach et al., 2003; Crighton et al., 2004, 2008), with a shift during puberty showing reproductive-age females experiencing the most severe outcomes (Seki et al., 2018; Wong et al., 2019). Studies indicate improved outcomes in male mice after treatment with exogenous testosterone, attributed to its anti-inflammatory effects (Vom Steeg et al., 2016). Immune cells, including microglia, express receptors for sex steroids, which can regulate gene expression and immune responses post-viral infection (Klein and Roberts, 2010). Microglia play a pivotal role in brain immune responses, synaptic remodeling, and plasticity. Activated microglia produce pro-inflammatory cytokines and chemokines and can potentially catalyze a prolonged overreaction of the inflammatory response (Perlmutter et al., 1992; Nakamura, 2002). In addition, microglia are important for synaptic remodeling and plasticity in the healthy brain (Parkhurst et al., 2013; for review see: Michell-Robinson et al., 2015). Upon IAV infection, microglia can engulf dysfunctional synapses (Wake et al., 2009), potentially leading to reduced spine density and synaptic plasticity in the hippocampus, ultimately leading to a decline in cognitive function (Hosseini et al., 2018). Microglia exhibit different morphologies based on their functions and activation status. Ramified microglia, characterized by numerous branches and high cellular complexity, interact dynamically with neighboring cells to maintain neurological homeostasis (Heneka et al., 2014). Amoeboid microglia predominantly phagocytose dysfunctional synapses and debris. Microglial activation and function are influenced by sex, as they express steroid hormone receptors (Nissen, 2017; Guneykaya et al., 2018). Additionally, antigen presentation as well as RNA and protein levels differ between male and female microglia (Guneykaya et al., 2018). Recent evidence suggests male and female microglia react differently to inflammatory insults, with female neonatal microglia showing less migration than male microglia upon stimulation (Yanguas-Casás et al., 2018). The mechanisms underlying the interplay between sex, neurotropic IAV infection, and subsequent microglial responses, in triggering neuroinflammation and potentially leading to neurodegeneration are not fully understood.

The aim of our study was to elucidate sex-specific responses of microglia and neurons to IAV infection. We used the polybasic influenza A strain rSC35M (recombinant A/Seal/Mass/1/80 mouse adapted, H7N7) (Gabriel et al., 2005) as a model for infection with a neurotropic IAV and primary hippocampal cultures including astrocytes, neurons, and microglia derived from either male or female mice.

Our results indicate a higher early infection rate in cultures containing microglia, suggesting that direct infection of microglia influences *in vitro* infection kinetics. In both sexes, neurotropic IAV infection induces spinogenesis, increasing the risk of neuronal network hyperactivation and subsequent excitotoxicity. In male-derived cultures, excessive spines appeared to be pruned directly by activated microglia. We hypothesize that hyper-ramified microglia, observed in both sexes, preferentially engulf presynaptic terminals, whereas amoeboid-shaped microglia, which increased only in male-derived cultures, may target postsynaptic terminals for phagocytosis. This, combined with the elevated profile of pro-inflammatory cytokines and chemokines in female-derived cultures post-IAV infection, suggests a higher neuroinflammatory status and an increased risk for neuropathological consequences in females.

## 2 Materials and methods

### 2.1 Preparation of poly-L-Lysine coated coverslips

For the preparation of poly-L-lysine-coated coverslips, glass coverslips with a diameter of 13 mm and a thickness of 1 mm were incubated in 10 M NaOH for 3–5 h at 100 °C. The coverslips were then washed five times with distilled water for 20 min. The coverslips were sterilized at 225 °C for 6 h. After cooling, the coverslips were coated with 0.5 mg/ml poly-L-lysine (Sigma-Aldrich, P2636) dissolved in boric acid buffer at 37°C for 2-3 h. The coverslips were washed five times with distilled water. After drying, they were stored at 4°C until further use.

### 2.2 Preparation of primary embryonic hippocampal cultures

Primary embryonic hippocampal cultures were prepared as previously described (Michaelsen et al., 2010). Briefly, C57BL/6J mice were decapitated at embryonic day 17.5 (E17.5) and embryos were divided by sex (Murdaugh et al., 2018). Hippocampi were separated from the cortex and dissociated in 1X trypsin/EDTA solution (Sigma-Aldrich, T3924) for 25 min at 37 °C and then homogenized by mechanical pipetting with a narrowed Pasteur pipette. Homogenized hippocampi were centrifuged for 5 min at 1500 rpm and the cell pellet resuspended in DMEM medium (Gibco, 6195-026) supplemented with 10% fetal calf serum (FCS) (Capricorn-scientific, FCS-62A) followed by three washes in neurobasal medium (Invitrogen, 21103049). Subsequently, 7 × 10^4^ cells per well were plated onto poly-L-lysine-coated 13 mm diameter coverslips in a 24-well plate and cultured for 21 days in neurobasal medium (Gibco, 21103-049) supplemented with N2 (autoclaved), B27 (Invitrogen, 17504-001) and 0.5 mM L-glutamine (Invitrogen, 25030-024) to obtain co-cultures of primary neurons and astrocytes. Co-cultures were maintained in an incubator at 37°C, 5% CO_2_ and 99% humidity, with 20% of the cell medium changed once a week.

### 2.3 Preparation of triple co-culture of neurons, astrocytes and microglia

Primary embryonic hippocampal cultures containing neurons and astrocytes were first prepared as described above. C57BL/6J mice were decapitated at postnatal day 3–5 (P3-5) and pups were seperated by sex (Wolterink-Donselaar, 2009). Cerebral cortices were harvested in cold 1X HBSS medium (Gibco, 14185-045) and centrifuged at 2000 rcf for 5 min at 4 °C. Cells were cultured for 14 days in DMEM medium (Gibco, 6195-026) containing 10% fetal calf serum (FCS) (Capricorn-scientific, FCS-62A) in poly-D-lysine (Sigma-Aldrich, 6407) coated 75 cm^2^ flasks (TPP, 90075) at 37 °C, 10% CO_2_, preserving only glial cells. Both cortical hemispheres of one pup were used per flask. Microglia were then isolated by shaking the flasks in a shaker incubator at 200 rpm for 1 h at 37 °C. 3 × 10^4^ microglia per well were plated on the embryonic hippocampal culture. Triple co-cultures were stored in an incubator at 37 °C, 5% CO_2_ and 99% humidity and treated 72 h after microglia plating.

### 2.4 Chemical transfection of primary cultures

To visualize individual neurons and dendritic spines, dissociated primary hippocampal cell cultures were transfected with a vector expressing pm-Citrine-C1 (L96, addgene, 54587). First, 1 μg L96 and separately 2 μl Lipofectamin^®^ 200 (Invitrogen, 52887) were diluted in 50 μl Neurobasal medium for each well of a 24-well plate. This solution was vortexed briefly and incubated for 5 min at room temperature. The solutions were then mixed to form the transfection solution, mixed again briefly and incubated for 20 min at room temperature. Meanwhile, 150 μl of cell culture medium per well was added to two free wells of each 24-well plate. At the end of incubation, 350 μl of medium was removed and retained in two free wells per plate. 100 μl of transfection solution per well was added dropwise to the cells and incubated for 50 min in an incubator (36.5 °C, 5% CO_2_, 99% humidity). The transfection solution was then replaced by the previously stored old medium, which was filled up to 500 μl per well with new medium. Cells were cultured for a further 3–4 days until infection.

### 2.5 Influenza A virus production and viral titer determination

Madin-Darby canine kidney (MDCK) cells were used for virus production and titration. Cells were cultured in DMEM (Life Technologies, 52100021) containing 10% fetal bovine serum (Sigma-Aldrich, F7524). For virus production, 90% of confluent Madin-Darby canine kidney (MDCK) cell monolayers were used. Cell culture medium was discarded, and the cells were washed three times with PBS (Gibco, 10010-015) before DMEM with 0.2% bovine serum albumin (BSA) (Sigma-Aldrich, A7409), 1% Penicillin/Streptomycin (Gibco, 15070-063) and 2.5 ng/ml NAT (Sigma-Aldrich, T6763) was added as infection medium. An adequate volume of either MOCK (PBS) or virus (kindly provided by Prof. Dr. Klaus Schugart) was added, and cells were incubated for 1 h at 37°C and 5% CO_2_. Afterward, the medium was discarded, the cells were washed with warm PBS containing 0.2% BSA (Sigma-Aldrich, 9048-46-8) and fresh infection medium was added. After approximately 40 h when a strong cytopathic effect was observable the supernatant was centrifuged at 4°C with 15000 rpm, the pellet was discarded and medium containing the virus was aliquoted and stored at −80°C until use. The virus was never thawed and frozen again.

Titres were determined by the FOCI assay in MDCK cells. For this, 3 × 10^4^ MDCK cells per well were plated on a 96-well plate in DMEM high glucose medium containing 10% FCS. Next day, a dilution series of virus samples in 10-time steps was prepared in FOCI infection medium. For FOCI infection medium, DMEM containing 0.1% BSA (Sigma-Aldrich, A7409) and 2.5 ng/ml NAT was used. The cell culture medium was discarded, and cells were washed once with infection medium before virus dilutions were added to each well and incubated for 1 h at 37°C in 5% CO_2_. Afterward, the infection medium was discarded and Cellulose-DMEM was added containing 50% 2xDMEM (2.68% DMEM (Life Technologies, 52100021), 0.75% NaHCO_3_ (Carl Roth, 6885.1), 2% Glutamax (Life Technologies, 35050061), 2% penicillin/streptomycin, adjusted with HCl (Carl Roth, 9277.1) to pH 7.2 and sterile-filtered using 0.2 μm unit (Sarstedt, 83.1826.001), 1% carboxymethylcellulose sodium (Sigma-Aldrich, C9481), 0.1% BSA and 2.5 ng/ml NAT in H_2_O. At 24 hpi the Cellulose-DMEM was discarded by tapping the well plate carefully. Cells were washed two times with PBS (self-made with Gibco, 18912-014), and cold 100% EtOH (J.T. Baker, 8025) was added for 10 min at room temperature to fixate the cells. Again, cells were washed two times with PBS, then Quencher [1.5% glycine (Serva, 23391.03)] and 0.5% Triton X100 (Sigma-Aldrich, T8787) in PBS was added for 10 min at room temperature and cells got washed once with washing buffer [0.5% Tween 20 (Sigma-Aldrich, 8.22184)] in PBS. The first (Virostat, 1301-1) and secondary antibody (KPL, 5220-0362) were each diluted 1:1000 in blocking buffer (0.5% Tween20, 1% BSA in PBS), incubated for 1 h at room temperature and added one after the other. After each antibody incubation, the cells were washed three times with PBS. In the end, cells were incubated for 15–30 min with peroxidase substrate (KPL, 5510-0030) and rinsed with water. Plaques were now visible and those dilutions with 3-30 foci per well were counted. The titer, foci forming units (FFU) per ml, was determined by multiplying the counts per well with the respective dilution factor and by dividing this number with the volume of diluted virus being added. For rSC35M a titer of 1 × 10^6^FFU/ml was acquired.

### 2.6 Infection of cultures with IAVs

Co- and triple co-cultures were infected with the influenza A virus H7N7 strain polybasic rSC35M (recombinant A/Seal/Mass/1/80 mouse-adapted) using 3500 FFU for co-culture infection and 4000 for triple co-culture infection, corresponding to a multiplicity of infection (MOI) of 0.05 for 70,000 and 80,000 cells per coverslip, respectively. The respective amount of virus was diluted in neurobasal medium (Invitrogen, 21103049) supplemented with N2 (custom-made), B27 (Invitrogen, 17504-001), and 0.5 mM L-glutamine (Invitrogen, 25030-024) (infection medium). As a control, the respective amount of DMEM with 10% FCS (Sigma-Aldrich, F7524) was used. To initiate the infection, the cell culture medium was exchanged with the infection medium. The cells were incubated for 1 h at 37 °C in 5% CO_2_ and afterward washed twice with cell culture medium. The cells were incubated for the required time at 37 °C in 5% CO_2_. At 6 and 24 hpi, the infection was stopped by fixation with 4% paraformaldehyde (PFA, Thermofisher scientific, 28908) for 15 min at room temperature. Cell culture supernatants were collected and stored at −80°C until further use.

### 2.7 Immunocytochemistry

Cells were fixated in 4% PFA (Thermofisher scientific, 28908) for 15 min at room temperature and afterward washed 3 times with self-made PBS for 5 min on a shaker. Subsequently, cells were permeabilized with 0.2% Triton X-100 (AppliChem, A4975,0100) for 30 min on a shaker and blocked in 1% BSA for 5 min to prevent non-specific binding. Afterward, cells were stained with primary antibodies and incubated for 1 h at 36 °C in a humidity box. Cells were washed 3 times and blocked again with 1% BSA for 5 min at room temperature on a shaker, which was followed by secondary antibody incubation for 30 min at 36 °C in a humidity box. Finally, nuclear DNA was counterstained with 1:1000 DAPI for 5 min at room temperature and washed three times with PBS and once with MilliQ water for 5 min on a shaker. Coverslips were mounted on glass slides in Fluorogel embedding medium (Electron Microscopy Sciences, Hatfield, PA).

For infection rate analysis the following antibodies were used: rabbit anti-NP (1:1000, GeneTex, GTX125989), chicken anti-NeuN (1:1000, Synaptic Systems, 266006) and mouse anti-GFAP (1:1000, Sigma-Aldrich, G3893) with a-rabbit Cy5 (donkey) (1:500, Jackson Immuno Research, 711-175-152), a-chicken Cy3 (donkey) (1:500, Milipore, AP194C) and a-mouse Cy2 (goat) (1:500, Jackson Immuno Research, 115-225-206). For microglial activation analysis the following antibodies were used: goat anti-IBA1 (1:1000, Novusbio, NB100-1028), rat anti-CD68 (1:1000, Invitrogen, 14-0681-82) and rabbit anti-Ki67 (1:1000, Abcam, ab16667), a-goat Cy2 (donkey) (1:500, Jacson Immuno Research, 705-225-147), a-rat Cy3 (donkey) (1:500, Jackson Immuno Research, 712-165-153) and a-rabbit Cy5 (donkey) (1:500, Jacson Immuno Research, 711-175-152). For analysis of synaptic terminals mouse anti-PSD95 (1:1000, invitrogen, MA1-045) and guineapig anti-synaptophysin (1:1000, Synaptic Systems, 101004) with a-mouse Cy5 (goat) (1:500, Jackson Immuno Research, 115-175-205) and a-guineapig Cy3 (goat) (1:500, Jackson Immuno Research, 706-166-148) were used.

### 2.8 Fluorescence microscopy

Images of immunocytochemical stainings were acquired using a wide-field fluorescence microscope (AxioImager 2 equipped with an ApoTome^®^ module, Zeiss). For the analysis of microglia morphology and activation status, z-stacks with an increment interval of 0.25 μm were acquired using the 20X objective (NA 0.8). Mean fluorescence intensity was analyzed using ImageJ [Fiji 1.54f, (Schindelin et al., 2012)]. The exposure time was kept constant for each staining to allow for direct comparison of expression levels.

For the analysis of dendritic spines, images of primary neurons expressing citrine and stained against PSD95 and synaptophysin were acquired using a Leica TCS SP8 STED microscope (HC PL APO CS2 93x oil objective, NA 1.30, pixel size 102 nm) and the Leica Application Suite X (LASX). A white light laser (WLL) with a tuning range of 470 to 670 nm was used at 85%. Additionally, a 592 nm STED laser, which was set to 70%, was used. For the anti-PSD-95 staining, an adsorption range of 655–779 nm was set and a hybrid detector (HyD) with a gaiting of 1.5–6 was used. Moreover, the emission laser was set to 649 nm. An adsorption range of 559–699 nm and an emission of 554 was set for the anti-synaptophysin staining. A hybrid detector (HyD) with a gaiting of 1.5–6 was used. For m-citrine, an adsorption range of 521–570 nm and an emission of 515 nm was set. A hybrid detector (HyD) with a gaiting of 1.5–6 was used. Additionally, the STED laser intensity was set to 75%.

### 2.9 Infection rate analysis

For analysis of the infection rate, a self-written segmentation code was used to determine the number of cells within the image using the DAPI channel and the number of infected cells using the nucleoprotein-stained channel. The channels were binarized utilizing the OpenCV library (version 4.8.1.78) with dynamic thresholding and the cell nuclei channel was prior processed with a Gaussian blur filter using the cv2.GaussianBlur function. Additionally, the principal of finding the sure foreground and background of the cells or nucleoprotein staining was performed to improve the thresholding (Bhattiprolu, 2020). Afterward, segmentation was performed using watershed segmentation. The czifile library (version 2019.7.2) was used for image handling, data handling was performed using pandas (version 2.1.4) and NumPy was used for data processing (version 1.26.2). The code is available upon request.

### 2.10 Microglial activity analysis

For analyzing the mean intensity of CD68 and IBA1 within microglia, the channel was first converted in a 2D image using the build-in maximum intensity z-projection. Afterward, the IBA1-channel was binarized in ImageJ [Fiji 1.54f, (Schindelin et al., 2012)] by applying a selected auto-threshold (Huang dark). If necessary, disconnected branches of microglia cells were manually reconnected. This step was performed by comparing the binary images with the original image. Then, the built-in analyze particles tool with a set minimum size of 100 was used to obtain the ROIs of each microglia cell within the image. Furthermore, each individual binary microglia cell was saved individually for morphological analysis. The obtained ROIs were used to measure the mean gray value of CD68 and IBA1 in each cell on the original image.

### 2.11 Morphological analysis

Changes in microglial morphology were analyzed as previously described (Fernández-Arjona et al., 2017). The branching data were obtained using the *MorphData* macro (Campos et al., 2021), which utilizes the *AnalyzeSkeleton* plugin (Arganda-Carreras et al., 2010) and processes the data for further analysis. The different morphological parameters (cell perimeter, convex hull perimeter, cell circularity, convex hull circularity, cell area, convex hull area, fractal dimension, lacunarity, density, roughness, convex hull span ratio, diameter of the bounding circle, maximum span across the convex hull, ratio maximum/minimum convex hull radii, mean radius) were analyzed using the *FracLac* plugin (Karperien, 2007-2012). Finally, a three-component principal component analysis (PCA) was performed by first scaling the data using the preprocessing scale function of the Sklearn library and then using the functions of the same library to fit the scaled data to obtain the coordinate data for the PCA graphs and further analysis (Sklearn version 1.4.0). The optimal number of clusters was selected using the elbow method, followed by K-Means clustering (Sklearn version 1.0.2) to categorize microglia cells according to the degree of dissimilarity of morphological parameters (fractal dimension, lacunarity, density, span ratio major_minor, area of convex hull, convex hull perimeter, convex hull circularity, diameter of bounding circle, average radius, maximum span of convex hull, max_min radii, area of cell, perimeter of cell, roughness, circularity of cell, number of branches, average length of branches, maximum length of branches). The matplotlib library was used to display the obtained data (matplotlib version 3.8.2). In addition, the NumPy (version 1.26.3) and pandas (version 2.2.0) libraries were used for data processing.

### 2.12 Dendritic spine analysis

Spine density analysis of transfected primary cultures was performed in Fiji [ImageJ 1.54f, (Schindelin et al., 2012)]. First, the length of each dendrite was measured using the segmented line tool and the measure function. Second, all spines of the dendrite were counted using the multipoint tool. Finally, the number of dendritic spines per μm for each dendrite was calculated. For analyzing the morphology of dendritic spines, spine head and neck coordinates (ROIs) as well as direct morphological features such as head area, spine and neck length were obtained using the *SpineJ* plugin (Levet et al., 2020). To access the neck and head coordinates, the source code of *SpineJ* was adapted in the NeuronDisplayDialog class. For analyzing the functional synapse, the PSD-95 and synaptophysin staining was further processed. First, the stacks were converted into 2D images using the built-in maximum intensity z-projection and the quality of the images were enhanced by first duplicating the images and applying the Unsharp Mask filter with a radius of 3 and a mask of 0.6 to one of the duplicates and subsequently using the Gaussian blur filter with a sigma of 25 to the other one. Afterward, the latter is subtracted from the first. Then, a selected auto-threshold (moments dark) is applied to binarize the images and the watershed algorithm is used. The analyze particles function with a minimum size of 0.06 is used to obtain ROIs of each synaptic terminal puncta and saved for later analysis. The obtained ROIs as well as the morphological characteristics obtained via SpineJ were used in a self-written python code to analyze the spine types and functional synapses within every given image. When the python code is launched, the user is asked to open a folder containing the above generated data. First, spine types are classified using the morphological spine data calculated by SpineJ (Levet et al., 2020) as follows: filopodia (spine length > 2 μm or head diameter/spine length < 0.25 μm and spine length > 1.5), mushroom (2 μm > spine length > 1 μm and head diameter > 0.6 μm and head diameter/neck diameter > 1), stubby (spine length < 1 μm) and thin (1 μm < spine length < 2 μm). Then the code checks if there is an overlap between the PSD-95 staining and the spine head area. When there is an overlap, the code calculates the intersection area. To be assigned as PSD, the intersection area can overlap up to 50% with the spine head area and has to overlap at least 10% with the PSD-95-stained area. Afterward, the code checks if the observed PSD belongs to a functional synapse via analyzing the intersection area with the synaptophysin staining. If the PSD-95 and synaptophysin dots overlap at least 10% the respective spine is categorized as spine with functional synapse. Finally, all obtained data are summarized by the code in a .csv file. The NumPy (version 1.26.3) and pandas (version 2.2.0) libraries were used for data processing. Furthermore, to load the ROI data obtained with ImageJ, the read-roi library (version 1.6.0) was used. For analyzing the intersection area, the shapely library (version 2.0.2) was used.

### 2.13 Enzyme-linked immunosorbent assay (ELISA)

Supernatants of co- and triple co-cultures 6 and 24 h infected with H7N7 IAV were collected and stored at –80°C until use. Mouse TNF-α DuoSet (DY410), mouse TGF-β (DY1679), mouse IL-1β/IL-1F2 DuoSet (DY401), mouse IL-6 (DY406), mouse IL-10 DuoSet (DY417), mouse IFN-β (DY8234-05), mouse CCL2/JE/MCP-1 DuoSet (DY479), and mouse CCL5/RANTES (DY478) ELISA kits (R&D Systems) were used to determine the levels of cytokines (TNF-α, TGF-β, IL-1β, IL-6, IL-10, IFN-β) or Chemokines (CCL2, CCL5) in the cell supernatants. In brief, 100 μl of cytokine-specific capture antibodies were applied to medium binding 96-well microplates and incubated overnight at room temperature. Plates were washed three times with 0.5% v/v Tween20 (Roth, 9127.2) in PBS and blocked in 300 μl 1% BSA (Sigma-Aldrich, A-7906) in PBS for 1 h at room temperature. After washing again, 100 μl of the samples and standards were added and incubated for 2 h at room temperature. Plates were then washed three times and incubated with 100 μl of detection antibodies for 2 h at room temperature. The plates were rinsed and 100 μl of streptavidin-HRP was added per well and incubated for 20 min at room temperature in the dark. After washing again, the substrate solution (R&D Systems, 642736) was added and incubated for 20 min, followed by 50 μl of the stop solution consisting out of 5% sulfuric acid (Applichem, 7664-93-9). The plates were read at 450 nm in a BioTek Synergy H1 microplate reader using Gen5 software (BioTek, USA). Finally, the measured optical density of the reaction was compared with the optical density of the known standard samples to determine the protein concentration in the samples.

### 2.14 Analysis using IMARIS

For the morphological analysis of the microglia, the software IMARIS 7.4.2 was used. First, the surface area and volume of the microglia were determined by setting the surface area detail level to 0.2 μm and the threshold to 8. For the measurement of the surface area and volume of lysosomes inside the microglia, the channel of LAMP1 was masked in the channel of IBA-1. Afterward, the surface area detail level was set to 0.2 μm and the threshold to 4. In the end of the analysis, the amount of pre- or postsynaptic terminals inside the microglia and inside the lysosomes in the microglia were determined. For this, the channel of synaptophysin or PSD95 was masked first in the channel of IBA-1 and subsequently in the masked channel of LAMP1 to detect the signals inside the lysosomes inside the microglia. At last, the estimated diameter of synaptic terminal puncta was set to 0.5 and the quality was set to 2.

### 2.15 Data visualization

Data visualization was performed with Graphpad Prism 7 (Graphpad Software, Inc, United States) and figures were prepared using Adobe Illustrator CS5 (Adobe Systems Incorporated, version 15.0.0). Furthermore, BioRender was used for the graphical abstract.

### 2.16 Statistical analysis

Statistical analysis was performed using Graphpad Prism 7 (Graphpad Software, Inc, United States). Data were presented as mean ± SEM. Differences between experimental groups were determined by two-way ANOVA, to analyze the effect of sex and infection as well as a potential interaction, followed by Tukey’s *post hoc* test for multiple comparisons. One-way ANOVA followed by Sidak’s *post hoc* test for multiple comparisons was used to analyze differences between triple co-cultures and co-cultures, as well as differences in the infection rate. The confidence interval was set to *p* < 0.05. The number of the different experimental groups is indicated in the respective figure legends. All experiments were evaluated in a strictly blind fashion. If applicable, normalization was performed in comparison to female controls.

## 3 Results

### 3.1 Microglial cells enhance viral propagation dynamics in a triple co-culture model

**FIGURE 1:**
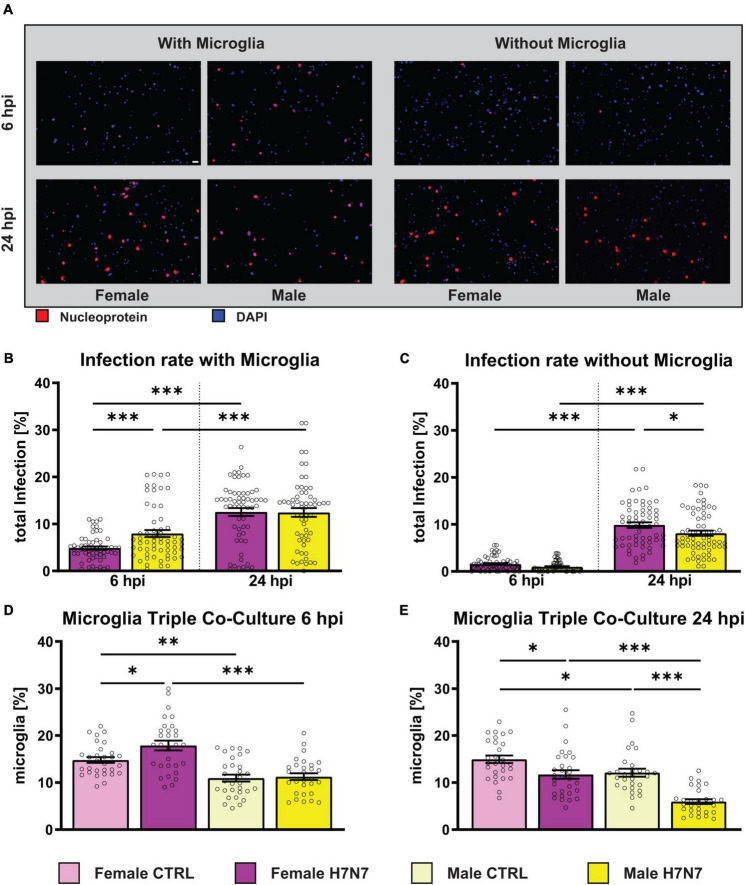
Total infection rate of primary hippocampal cultures derived from females and males after infection with influenza A/Seal/Mass/1/80 rSC35M (H7N7) virus. **(A)** Examples of images showing the amount of virus entering neuronal cells as indicated by immunostaining with influenza nucleoprotein (NP, red) and DAPI staining (blue). Scale bar: 20 μm. **(B)** Infection rate of female- and male-derived cultures with microglial involvement. A FFU of 4000 was used. **(C)** Infection rate of female- and male-derived cultures without microglial involvement. A FFU of 3500 was used. *N* = 4, *n* = 20 images per group and cell preparation round. **(D)** The percentage of female-derived microglia increased at 6 hpi. **(E)** A decrease, especially in male-derived microglia, was observed at 24 hpi. Number of experiments, *N* = 3, *n* = 10 images per group and cell preparation round. Data are presented as mean ± SEM and infection rate data were analyzed by one-way ANOVA followed by *post hoc* Tukey test. *p < 0.05, **p < 0.01, and ***p < 0.001.

IAV infection results in more severe outcomes in females of reproductive age compared to males (Seki et al., 2018; Wong et al., 2019). To investigate sex-specific differences in cell types of the central nervous system (CNS) upon IAV infection, we utilized primary hippocampal cultures, both without and with the presence of microglia, prepared from female and male mice, here after referred to as co-cultures and triple co-cultures, respectively. We used a neurotropic H7N7 subtype (recombinant A/Seal/Mass/1/80 mouse-adapted) with a FFU of 3500 and 4000 for infection analysis of co-cultures and triple co-cultures, respectively. The infection rate was analyzed 6 and 24 h post infection (hpi) to assess initial viral infection and replication dynamics, as seen in cell-to-cell spread (Figure 1, means with SEM in Supplementary Table 1).

Infection rates of female- and male-derived triple co-cultures (Figure 1B) significantly increased at 24 hpi compared to 6 hpi (one-way ANOVA: *F*_(3, 232)_ = 24.15, *P* < 0.0001, female: *p* < 0.0001, male: *p* = 0.0002). Furthermore, a significantly higher infection rate was observed in male-derived cultures compared to female-derived cultures at 6 hpi (*p* = 0.0171). To investigate the role of microglial during infection, cultures without microglia were also examined (Figure 1C). The initial infection rate at 6 hpi was lower in the absence of microglia (one-way ANOVA: *F*_(3, 250)_ = 115.5, *P* < 0.0001, female_6 hpi_: *p* < 0.0001, female_24 hpi_: *p* < 0.0021, male_6 hpi_: *p* < 0.0001, male_24 hpi_: *p* < 0.0001). As observed in microglia-containing cultures, the number of infected cells increased significantly at 24 hpi (female: *p* < 0.0001, male: *p* < 0.0001). A comparison between the sexes revealed more infected cells in female-derived cultures compared to male-derived cultures at 24 hpi (*p* = 0.0129). The results indicate that H7N7 virus replication in the cultures is independent of the presence of microglia and reveal sex-specific differences in infection rates that vary with the presence of microglia. Interestingly, analysis of the total number of microglia showed a significant increase in microglia only in female-derived cultures at 6 hpi (*p* = 0.0247, Figure 1D). The percentage of microglia decreased significantly in both male and female cultures at 24 hpi (male: *p* < 0.0001, female: *p* = 0.0163, Figure 1E), although the percentage of microglia remained significantly lower in male than in female cultures (*p* < 0.0001). Notably, the percentage of microglia in female-derived cultures was higher in both the 6 h control (*p* = 0.0028) and the 24 h control (*p* = 0.0461) compared to male-derived cultures.

### 3.2 Sex-dimorphic cytokine and chemokine secretion profiles following H7N7 viral infection

Peripheral immune cells, along with microglia, astrocytes, and neurons within the CNS secrete pro-inflammatory cytokines and chemokines (Galic et al., 2012). The production of these pro-inflammatory mediators in the brain is a critical mechanism underlying many pathological conditions. While these molecules can initially help to direct and modulate the immune response, their excessive concentration and prolonged release can adversely affect neuronal cells and lead to immunopathogenesis (Hosking and Lane, 2010; Ramesh et al., 2013; DiSabato et al., 2016). IAV infection can induce the secretion of pro-inflammatory cytokines and chemokines (Jang et al., 2009, 2012; Bohmwald et al., 2021). Given that the immune response is influenced by the sex, we investigated the secretion of pro-inflammatory cytokines and chemokines in female- and male-derived cultures. Additionally, to explore the involvement of microglia in the secretion of these molecules, we performed the experiment in cultures with or without microglia.

**FIGURE 2:**
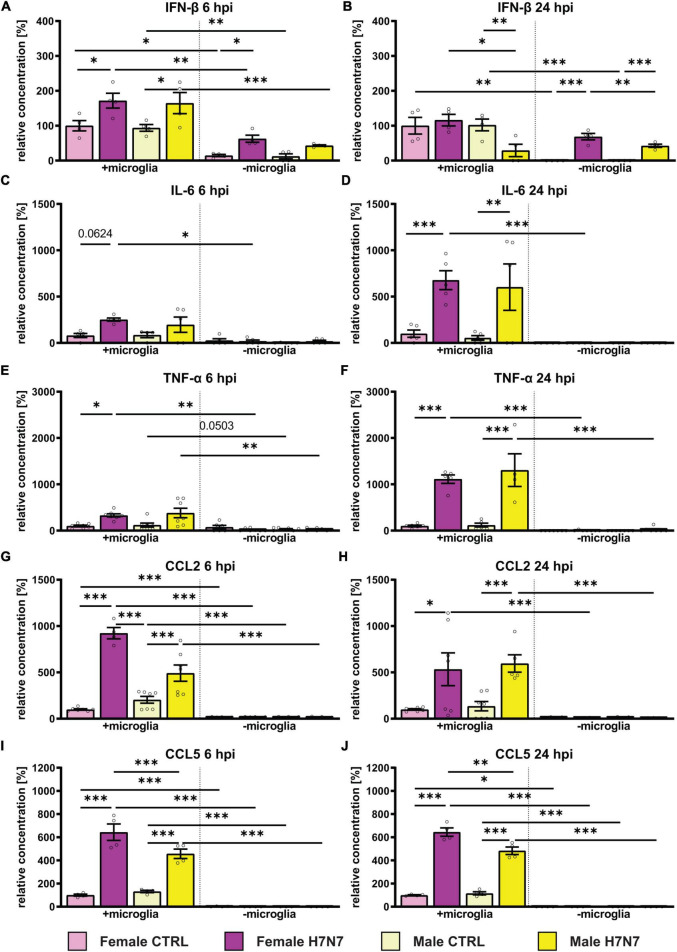
Effect of H7N7 IAV infection on the release of pro-inflammatory cytokines and chemokines in a sex-specific manner. **(A)** INF-β secretion increased after H7N7 infection at 6 hpi. **(B)** As a fast-responding interferon, IFN-β secretion decreased as early as 24 hpi and even decreased in male cultures after infection. **(C,D)** IL-6 secretion increased especially at 24 hpi in a microglia-dependent manner. **(E,F)** TNF-alpha secretion increased especially 24 hpi after infection in a microglia-dependent manner. **(G,H)** CCL2 secretion increased in a microglia-dependent manner at 6 and 24 hpi. **(I,J)** CCL5 secretion increased at 6 and 24 hpi in a microglia-dependent manner. Pro-inflammatory chemokine secretion was greater in female-derived cultures after infection. Number of experiments, *N* = 3, *n* = 2–3 replica per group and experimental round. Data are presented as mean ± SEM and were analyzed by two-way ANOVA followed by *post hoc* Tukey test. *p < 0.05, **p < 0.01, and ***p < 0.001.

Our results demonstrate an increase in the secretion of interferon-β (IFN-β) in both female- and male-derived cultures, which was more pronounced in cultures containing microglia. The response was rapid, with INF-β levels increasing as early as 6 h after infection (Figure 2A). The elevated IFN-β levels decreased to control levels by 24 hpi in female-derived cultures containing microglia, whereas IFN-β levels decreased significantly even below control levels in male-derived cultures (Figure 2B). In cultures without microglia, IFN-β levels remained high in both sexes at 24 hpi. Results of the respective two-way ANOVAs of Figure 2 can be seen in Supplementary Table 2.

Additionally, we observed a microglia-dependent increase in interleukin-6 secretion in female-derived cultures at 6 hpi (Figure 2C). IL-6 secretion further increased 24 hpi in a microglia-dependent manner in both sexes (Figure 2D). In cultures lacking microglia, the IL-6 concentration in the medium was low. Secretion of tumor necrosis factor alpha (TNF-α) at 6 hpi increased significantly only in microglia-containing cultures derived from females (Figure 2E). At 24 hpi we detected a significant microglia-dependent increase in TNF-α secretion in both sexes (Figure 2F).

The secretion of pro-inflammatory chemokines was only visible in cultures containing microglia, consistent with microglia being the main secreting cells. CC-Chemokinligand 2 (CCL2) secretion was significantly increased in male- and female-derived cultures containing microglia at 6 hpi and 24 hpi and unchanged without microglia involvement (Figures 2G, H). Secretion of CC-Chemokinligand 5 (CCL5) was significantly increased in female-and male-derived tri-cultures 6 and 24 hpi (Figures 2I, J). Moreover, CCL5 secretion was significantly higher in female cultures compared to males at 6 hpi (*p* < 0.0001) and 24 hpi (*p* = 0.0016).

In summary, infection with the H7N7 IAV subtype stimulated the secretion of pro-inflammatory cytokines and chemokines in a microglia-dependent manner, with a higher secretion of CCL2 and CCL5 as well as IFN-β in female-derived cultures. Furthermore, IFN-β secretion was also induced in the absence of microglia, indicating the involvement of astrocytes.

### 3.3 *In vitro* H7N7 viral infection induces morphological alterations in microglial cells

Microglia can undergo morphological changes depending on their activation status. Specifically, they transition from a complex branched ramified state to an intermediate reactive phenotype and, ultimately, to a roundish amoeboid state during viral infection (Streit et al., 2004; Sahu and Ter, 2018; Li et al., 2019; Edler et al., 2021). Furthermore, sex-specific differences in microglial morphology have previously been observed, with male microglia exhibiting a more complex phenotype (Yanguas-Casás, 2020; Han et al., 2021; Lynch, 2022). To investigate sex-specific alterations in microglia morphology after IAV infection, we infected female- and male-derived primary hippocampal cultures with the H7N7 substrain for 6 and 24 h. Morphological features were analyzed using the MorphData (Campos et al., 2021) and FraLac (Karperien, 2007-2012) plugin in ImageJ, followed by Principal Component Analysis (PCA) and K-Means clustering to categorize morphological subtypes into (hyper-)ramified, intermediate, and amoeboid microglia.

**FIGURE 3:**
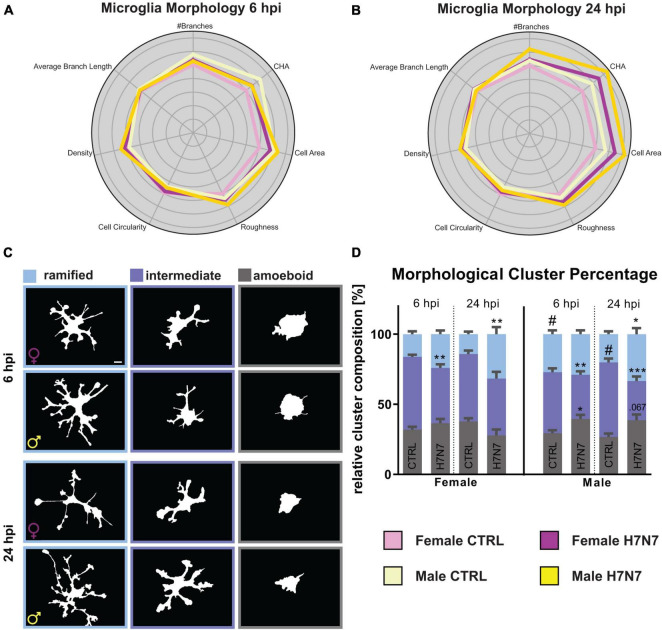
Microglia undergo morphological changes upon neurotropic IAV infection. **(A,B)** Radarplot displaying changes in morphological characteristics, namely number of branches, convex hull area (CHA), cell area, roughness, cell circularity, density, and average branch length at 6 and 24 hpi. The data are normalized to the female controls. A shift toward characteristics associated with hyper-ramified microglial morphology is observable in both sexes 24 hpi. An additional shift to amoeboid-shaped characteristics is observable in male-derived microglia. **(C)** Example images of binarized microglia. Scale bar: 5 μm. **(D)** Percentage of microglial morphological types change after infection with the neurotropic H7N7 IAV to a hyper-ramified phenotype in both sexes. Additionally, an increase in amoeboid-shaped microglia is observed. Number of experiments, *N* = 3, *n* = 10 images per group and cell preparation round. Data are presented as mean ± SEM and were analyzed by two-way ANOVA followed by *post hoc* Tukey test. *p < 0.05, **p < 0.01, and ***p < 0.001.

Analysis revealed that IAV infection increased cell complexity (number of branches, roughness, density), cell size (cell area, convex hull area), and cell circularity at 6 hpi (Figure 3A). These morphological changes were even more pronounced at 24 hpi (Figure 3B). Additionally, male-derived microglia appeared to be more complex and larger compared to female-derived microglia. To further analyze morphological alterations, we categorized microglia into the three previously defined subtypes. Amoeboid microglia exhibited reduced complexity and increased roundness following infection (Supplementary Figure 1), whereas ramified microglia became more complex and less roundish after infection, suggesting a transition to a hyper-ramified state (Supplementary Figure 2). Intermediate microglia initially increased in both amoeboid and ramified features at 6 hpi, but transitioned to more ramified features by 24 hpi, indicating a shift toward ramification (Supplementary Figure 3). To explore population dynamics, we performed K-Means clustering and quantified the proportion of each subtype (Figure 3D, mean values with SEM in Supplementary Table 1). Our data revealed a significant increase in the relative number of amoeboid microglia only in male-derived cultures at 6 hpi (Infection: *F*_(1, 113)_ = 8.014, *P* = 0.0055) accompanied by a decrease in intermediate microglia (Infection: F_(1, 113)_ = 26.17, *P* < 0.0001; Sex: *F*_(1, 113)_ = 12.16, *P* = 0.0007). A tendency toward increased ramified microglia was observed at 6 hpi (Infection: *F*_(1, 113)_ = 3.616, *P* = 0.0598). Overall, male-derived cultures contained more ramified microglia compared to female-derived cultures (Sex: *F*_(1, 113)_ = 9.696, *P* = 0.0023). At 24 hpi, the population of amoeboid microglia increased in male-derived cultures, while female-derived microglia became less amoeboid-shaped (Sex x Infection: *F*_(1, 114)_ = 10.25, *P* = 0.0018). This was consistent with a decrease in intermediate microglia after infection, predominantly in male-derived cultures (Infection: *F*_(1, 114)_ = 22.74, *P* < 0.0001, Sex x Infection: *F*_(1, 114)_ = 6.736, *P* = 0.0107). The relative number of ramified microglia increased in both sexes at 24 hpi (Infection: *F*_(1, 114)_ = 18.40, *P* < 0.0001). These results, along with observed increases in complexity post-infection, suggest hyper-ramification induced by IAV infection.

**FIGURE 4:**
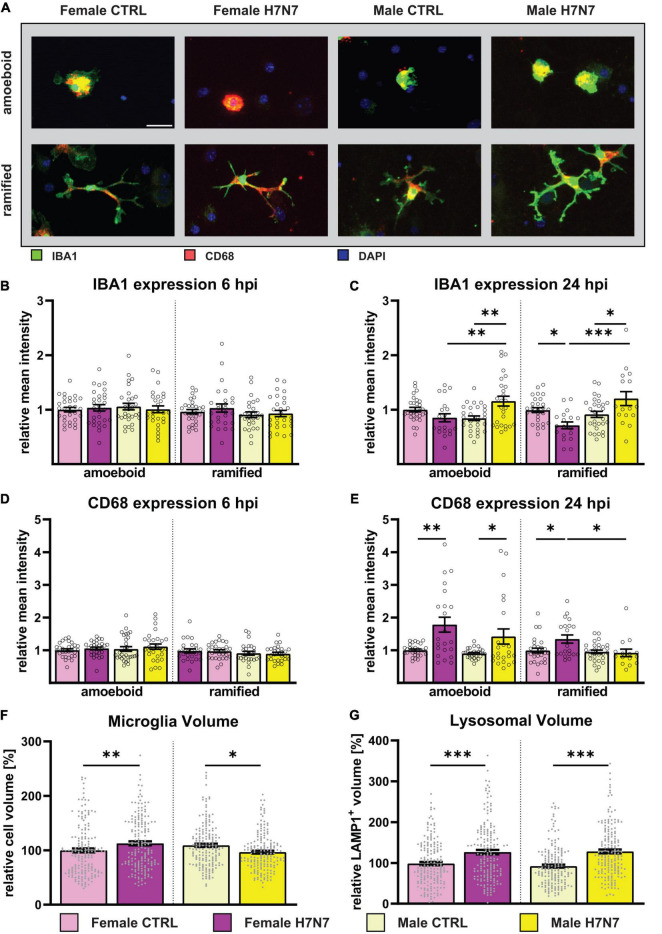
Microglial reactivity was induced by H7N7 virus infection *in vitro* **(A)** Example images displaying female-and male-derived amoeboid and (hyper-)ramified microglia after IAV infection. Green: IBA1, Red: CD68, Blue: DAPI. Scale bar: 20 μm. **(B,D)** No difference in microglial reactivity was observed 6 hpi. **(C)** An increase in IBA1 expression, indicating an increase in microglial reactivity, was observed in male-derived cultures in both amoeboid-and hyper-ramified microglia at 24 hpi. Contrarily, female-derived hyper-ramified microglia showed a decrease in IBA1 expression. **(E)** An increase in CD68 expression was observed in female- and male-derived amoeboid microglia and female-derived (hyper)ramified microglia at 24 hpi. Number of experiments, *N* = 3, *n* = 10 images per group and cell preparation round. **(F)** An increase in microglial cell volume in female-derived cultures was observed at 24 hpi, whereby a decrease was seen in male-derived cultures. **(G)** In both female- and male-derived cultures, an increase in the lysosomal volume was seen at 24 hpi indicating an increase in the phagocytotic activity. Number of experiments, *N* = 6, *n* = 30 cells per group and cell preparation round. Data are presented as mean ± SEM and were analyzed by two-way ANOVA followed by *post hoc* Tukey test. *p < 0.05, **p < 0.01, and ***p < 0.001.

We also analyzed microglia volume using IMARIS software, which showed an overall increase in microglia volume at 6 hpi, especially in female-derived microglia (Infection: *F*_(1, 701)_ = 12.04, *P* = 0.0006, Supplementary Figure 6A). Microglial cells in female-derived cultures exhibited increased volume at 24 hpi, consistent with observed hyper-ramification, while microglia in male-derived cultures showed a decrease in volume, aligning with the increased proportion of amoeboid cells (Infection: *F*_(1,707)_ = 15.82, *P* < 0.0001, Figure 4F).

### 3.4 H7N7 viral infection induces activation of microglial cells

As part of our continued investigation, we further characterized microglia after IAV infection by quantifying the expression of activation marker molecules. Immunocytochemistry was performed using antibodies against ionized calcium-binding adapter molecule 1 (IBA1) and CD68. IBA1 is involved in reorganizing the actin cytoskeleton by cross-linking actin to induce membrane ruffling (Sasaki et al., 2001), which is essential for microglial motility and phagocytosis (Oshawa et al., 2000). CD68 acts as a lysosomal marker, indicating phagocytic activity and thereby functional activation of microglia (Walker and Lue, 2015). Lysosomes are dynamic organelles of cellular catabolism, whose number, size, and distribution adapt to environmental cues (de Araujo et al., 2020).

At 6 hpi, we found no significant differences in the expression of IBA1 or CD68 (Figures 4B–D). The results of the respective two-way ANOVAs of Figure 4 are shown in Supplementary Table 2. However, at 24 hpi, an increase in IBA1 expression was observed in both amoeboid and ramified microglia in male-derived cultures (Figure 4C). In contrast, female-derived amoeboid microglia showed a significant decrease in IBA1 expression in ramified microglia at 24 hpi, whereas no differences were observable in amoeboid microglia.

IAV infection differentially affected CD68 expression as well. At 24 hpi, an increase was observed in amoeboid microglia derived from both sexes (Figure 4E), and only female-derived ramified microglia also showed an increase in CD68 expression at 24 hpi.

Given that most phagocytosed components are degraded in lysosomal vesicles, we next analyzed the total volume of lysosomes in microglia as an indicator of phagocytic activity. This was achieved through immunocytochemistry against Lamp-1 followed by volumetric analysis using IMARIS software (Figure 4G). A significant increase in lysosomal volume was observed in both sexes at 24 hpi, corroborating increased phagocytic activity.

Furthermore, we examined volumetric changes in microglial cells to validate our earlier findings of hyper-ramification in both sexes and an increased number of amoeboid microglial cells in male cultures at 24 hpi. Consistent with our conclusions, volumetric analysis showed an increase in cell volume in female-derived cultures and a decrease in cell volume in male-derived cultures at 24 hpi (Figure 4F).

### 3.5 Sex-specific modulation of spine density following H7N7 viral infection

To investigate the neuropathological consequences of IAV infection and resultant microglial activation, we analyzed spine density. Dendritic spines, which host the majority of excitatory synapses, are critical for neuronal connectivity. Previous *in vivo* studies have shown that non-neurotropic IAV infection leads to early-stage (7 days post-infection) spine changes in the hippocampus, including spine loss in regions such as the medial dentate gyrus, resulting in impaired cognitive function (Jurgens et al., 2012). Furthermore, long-term impairments in cognitive function following neurotropic H7N7 IAV infection and a reduction in spine density 30 dpi were reported (Hosseini et al., 2018). Notably, Hosseini et al. (2018) focused exclusively on female mice, leaving the impact on male individuals unexplored. Additionally, the immediate effects of neurotropic IAV infection on dendritic spine density remain unclear.

**FIGURE 5:**
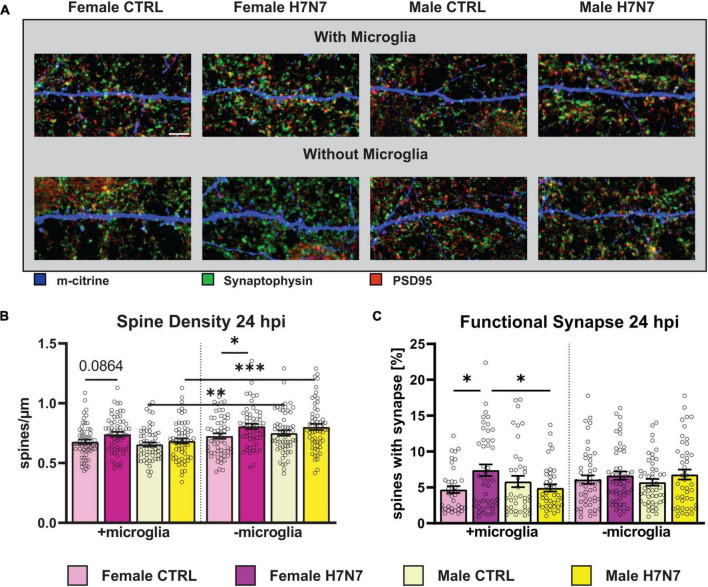
Changes in the number and morphology of dendritic spines as well as in the number of functional synapses during viral infection can lead to detrimental neurological consequences. **(A)** Example images displaying dendritic spines (blue), presynaptic (green) and postsynaptic terminals (red). Scale bar: 5 μm. **(B)** Number of dendritic spines increased in female-derived cultures independent of microglia presence at 24 hpi. **(C)** Amount of functional synapses increased in female-derived cultures in a microglia-dependent manner at 24 hpi. Number of experiments, *N* = 3, *n* = 20 images per group and cell preparation round. Data are presented as mean ± SEM and were analyzed by two-way ANOVA followed by *post hoc* Tukey test. *p < 0.05, **p < 0.01, and ***p < 0.001.

Our results revealed an increase in spine density at 6 hpi in cultures with and without microglia derived from both sexes (two-way ANOVA, Infection: *F*_(1, 236)_ = 3.232, *P* = 0.0735, Supplementary Figure 4B). Interestingly, at 24 hpi, spine density remained significantly elevated in female-derived cultures compared to the control, irrespective of microglia presence (Infection: *F*_(1, 234)_ = 5.640, *P* = 0.0184, Figure 5B). In male-derived cultures, however, spine density did not remain increased in cultures containing microglia but remained elevated in cultures without microglia (Sex: *F*_(1, 234)_ = 4.228, *P* = 0.0409, Infection: *F*_(1, 230)_ = 7.915, *P* = 0.0053), one-way ANOVA male with vs. without microglia: *F*_(3, 235)_ = 9.010, *P* < 0.0001). All mean values with SEM can be seen in Supplementary Table 1.

Next, we examined the proportion of functional excitatory synapses located on dendritic spines. Notably, the density of functional synapses increased at 6 hpi in female-derived cultures containing microglia (Infection: *F*_(1, 163)_ = 5.562, *P* = 0.0195, Supplementary Figure 4C) (Sex: *F*_(1, 163)_ = 4.778, *P* = 0.0302). In contrast, a significant decrease in functional synapse density was observed in cultures derived from both sexes without microglia (Infection: *F*_(1, 180)_ = 20.64, *P* < 0.0001). The overall number of functional synapses was higher in cultures with microglial involvement (*F*_(7, 343)_ = 5.857, *P* < 0.0001). At 24 hpi, a significant increase in the density of functional synapses was observed in female-derived cultures containing microglia (Figure 5C, *F*_(1, 204)_ = 6.712, *P* = 0.0103), an effect not found in cultures without microglia.

### 3.6 IAV infection modulates spine subtype composition

**FIGURE 6:**
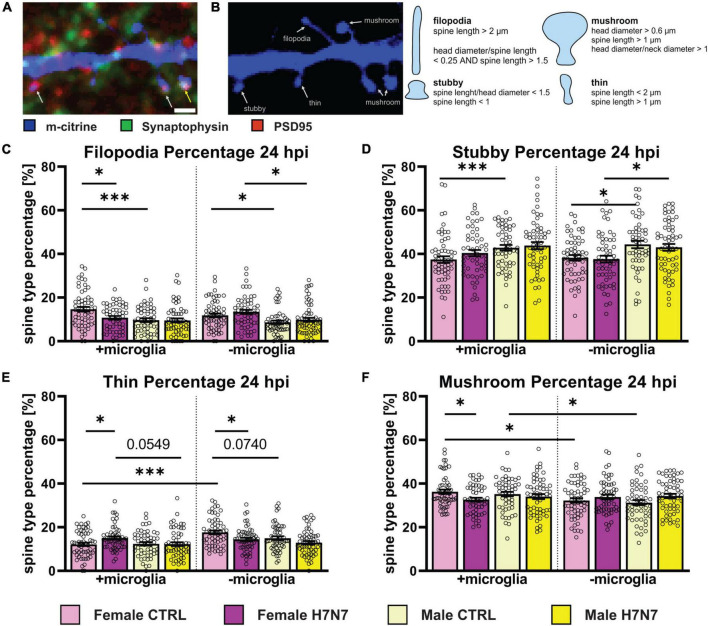
The morphology of dendritic spines can indicate their functional status. **(A)** Example image of a dendritic cutout displaying different morphological spine types (blue). In combination with an immunostaining for presynaptic (green) and postsynaptic terminals (red), spines with postsynaptic density (PSD) are highlighted with a white arrow and functional synapses, indicated by an intersection of post-and presynaptic terminals, are shown with a yellow arrow. Scale bar: 1 μm. **(B)** Example Image shows different spine types: filopodia, stubby, thin, and mushroom. Overview about morphometrical characteristics to categorize spines within the different spine types. **(C)** Filopodia percentage is decreased in female-derived cultures in a microglia dependent manner at 24 hpi. Female-derived cultures possess a higher filopodia percentage compared to male-derived cultures independent of microglia presence. **(D)** Stubby spine percentage is higher in male-derived cultures compared to female-derived cultures in a microglia-independent manner. **(E)** The proportion of thin spines is increased in female-derived cultures after neurotropic IAV infection in the presence of microglia and decreased in their absence. Furthermore, thin spine frequency was higher in female-derived cultures without microglia presence and generally higher in female-derived cultures compared to male-derived cultures independent of microglia involvement. **(F)** A decrease in mushroom spines was observed in female-derived cultures at 24 hpi. Baseline level of mushroom type spines was higher in a microglia dependent manner. Number of experiments, *N* = 3, *n* = 20 images per group and cell preparation round. Data are presented as mean ± SEM and were analyzed by two-way ANOVA followed by *post hoc* Tukey test. *p < 0.05, ***p < 0.001.

Dendritic spines play a crucial role in neuronal circuits by compartmentalizing biochemical and electrical signals in response to synaptic activation as they are highly plastic (Yuste, 2013). Spines contain neurotransmitter receptors within a membrane region known as the postsynaptic density (PSD) located in the spine head. These membrane protrusions can be classified into several subtypes: filopodia, which are considered precursors to spines; thin spines, which have smaller or no synapses on average; stubby spines, which lack an obvious neck region; and mushroom spines, which are regarded as prototypical mature spines with fully functional synapses (Peters and Kaisermann-Abramof, 1970; Bourne and Harris, 2007), shown in Figure 6B.

We analyzed the relative distribution of spine types at 6 hpi (Supplementary Figure 4) and 24 hpi (Figure 6). Notably, significant changes upon infection were only detectable in female-derived cultures, predominantly in cultures containing microglia. Female-derived cultures showed a decrease in filopodia only when microglial were present at 24 hpi (Figure 6C, Infection: *F*_(1, 218)_ = 4.580, *P* = 0.0335, Sex x Infection: *F*_(1, 218)_ = 3.951, *P* = 0.0481). Furthermore, a decrease in mushroom type spines in female-derived cultures containing microglia was observed at 24 hpi (Figure 6F, Infection: *F*_(1, 216)_ = 5.540, *P* = 0.0195). In addition, an increase in the percentage of thin spines was observed in microglia presence in female-derived cultures (Figure 6E, Infection: *F*_(1, 217)_ = 4.088, *P* = 0.0444, Sex x Infection: *F*_(1, 217)_ = 4.732, *P* = 0.0307). In contrast, cultures without microglia exhibited a decrease in thin spine density at 24 hpi (Infection: *F*_(1, 223)_ = 11.33, *P* = 0.0009, Sex: *F*_(1, 223)_ = 7.180, *P* = 0.0079). These observations indicate microglia-dependent pinocytosis of dendritic spine membranes, primarily affecting functional mushroom type spines, consistent with a decrease in spine head area of mushroom spines containing a synapse at 6 hpi (Supplementary Figure 5C, Sex x Infection: *F*_(1, 135)_ = 5.561, *P* = 0.0198). In contrast, no morphological changes were observed in male-derived cultures at 24 hpi.

### 3.7 H7N7 viral infection promotes engulfment of excitatory synapses by microglia

**FIGURE 7:**
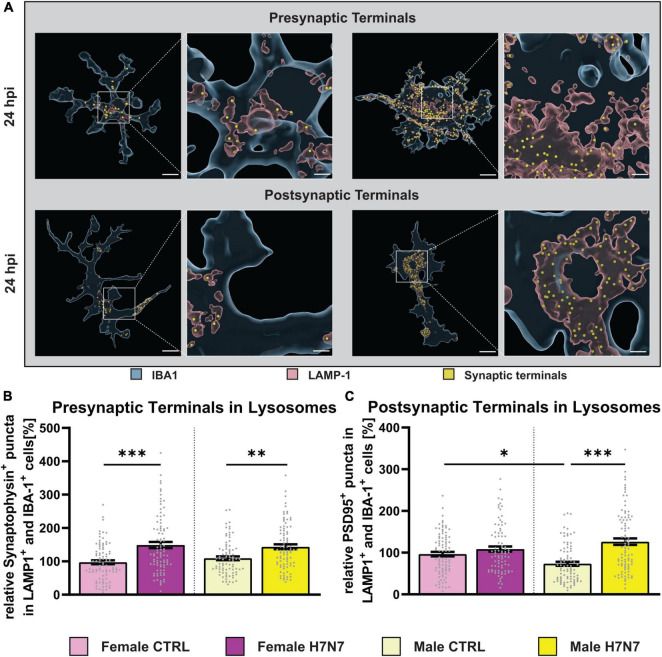
Engulfment of excitatory synaptic terminals after infection with neurotropic IAV H7N7. **(A)** Example images displaying phagocytosed pre-or postsynaptic terminals (yellow) within the lysosomes (red) of microglial cells (blue). **(B)** An increased engulfment of presynaptic terminals at 24 hpi was observed in female-and male-derived cultures. **(C)** Only in male-derived cultures, an increased engulfment of postsynaptic terminals was seen. Number of experiments, *N* = 3, *n* = 30 cells per group and cell preparation round. Data are presented as mean ± SEM and were analyzed with an ordinary two-way ANOVA with *post hoc* Tukey test. *p < 0.05, **p < 0.01, and ***p < 0.001.

Next, we sought to elucidate how microglia cells contribute to neuronal dysfunction following H7N7 infection. Previous studies have shown that activated microglia can disrupt homeostatic functions and induce neuronal damage, thereby affecting synaptic connectivity (Lima et al., 2022). Thus, neuronal damage caused by H7N7 infection could be mediated by microglial engulfment of synaptic terminals via phagocytosis or pinocytosis. To investigate this, we quantified the number of presynaptic (synaptophysin-positive puncta, Figure 7B) and postsynaptic (PSD95-positive puncta, Figure 7C) terminals within microglial lysosomes.

An increase in the number of engulfed presynaptic terminals was observed at 6 hpi (Infection: *F*_(1, 352)_ = 10.09, *P* = 0.0016, Supplementary Figure 6A) and 24 hpi (Infection: *F*_(1, 348)_ = 35.95, *P* < 0.0001, Figure 7B) in both sexes. No significant differences were observed in the number of engulfed postsynaptic terminals at 6 hpi (Supplementary Figure 6D). However, a significant increase in the number of engulfed postsynaptic terminals was detected at 24 hpi only in male-derived microglia (Infection: *F*_(1, 347)_ = 27.45, *P* < 0.0001, Infection x Sex: *F*_(1, 347)_ = 10.88, *P* = 0.0011). Furthermore, under control conditions, male-derived microglia already exhibited a lower number of engulfed postsynaptic terminals compared to female-derived cells (*p* = 0.0424). The number of engulfed postsynaptic terminals significantly increased at 24 hpi compared to 6 hpi in both sexes (*F*_(7, 701)_ = 14.39, *P* < 0.0001). In summary, our findings demonstrate that synaptic integrity is compromised by microglial activity in a sex-specific manner following H7N7 infection.

## 4 Discussion

Our study investigated the profound health risks posed by neurotropic influenza viruses, particularly the H7N7 subtype, which can infiltrate the central nervous system, causing neuroinflammation and a range of neurological complications. Notably, emerging evidence highlights sex-specific differences in the severity and manifestation of these neurological impairments, underlining the necessity for detailed studies on sex-specific responses to neurotropic viral infections. This study focused on the influence of sex on cellular responses following neurotropic IAV infection and explored how microglial cells contribute to neuronal network damage.

A deeper understanding of the kinetics of viral entry and replication in different CNS cell types is crucial for unraveling the intricate details of the infection process and identifying potential intervention strategies. Our findings significantly contribute to this understanding by showing that primary hippocampal cultures from both sexes exhibit a higher infection rate in the presence of microglia as early as 6 hpi. This accelerated infection rate is attributed to a higher infection level in microglia, which may lead to enhanced viral replication. Thus, microglia serve as initial targets for neurotropic infection with H7N7, altering replication kinetics and increasing the infection rate during early stages. Interestingly, female-derived cultures exhibited increased microglial proliferation at 6 hpi, likely triggered by viral entry, whereas male-derived cultures did not, indicating a sex-specific difference in the microglial response to viral infection. Furthermore, both sexes showed a decrease in microglia numbers at 24 hpi, potentially due to excessive inflammation or direct infection. Future studies are needed to investigate these mechanisms in more detail and to elucidate the pathological pathways involved in neurotropic IAV neurovirulence.

IAV infection is known to trigger the release of pro-inflammatory cytokines and chemokines, which can contribute to a “cytokine storm” (Jang et al., 2009, 2012; Bohmwald et al., 2021). Considering the influence of sex on immune responses, we investigated the secretion of these inflammatory mediators from CNS cell types in cultures derived from both female and male mice. Type-I interferons, such as IFN-β, play a vital role in the rapid and broad response to influenza A virus infection by inducing antiviral defenses (Shim et al., 2017; Muñoz-Moreno et al., 2021). Our data show a rapid increase in IFN-β secretion immediately after IAV infection, underscoring the validity of our cell culture model. In cultures without microglia IFN-β levels already declined by 24 hpi. Interestingly, male-derived cultures showed a reduction in IFN-β levels below control levels at 24 hpi, indicating a sex-specific interferon response to infection which was suggested earlier (Pujantell and Altfeld, 2022). In the absence of microglial involvement, we observed lower but prolonged IFN-β secretion, likely driven by astrocytes. Both male- and female-derived cultures exhibited increased levels of pro-inflammatory cytokines in a microglia-dependent manner, highlighting the key role of microglia as producers of pro-inflammatory cytokines in the CNS. Additionally, a microglia-dependent pro-inflammatory chemokine response was observed in both sexes, with a more pronounced effect in female-derived cultures. The C-C chemokine receptor type 5 (CCR5), which binds ligands like CCL5, has emerged as a key element in modulating network function through synaptic pruning, potentially contributing to observed learning and memory impairments (Guyon et al., 2005; Heinisch and Kirby, 2010). These findings support the hypothesis that females may be more susceptible to the neuropathological consequences of IAV infection. Indeed, *in vivo* studies have reported higher pro-inflammatory cytokine and chemokine release in females upon IAV infection (Larcombe et al., 2011). Larcombe et al. (2011) also concluded that host-mediated immunopathology, rather than viral replication, underlies sex-specific differences in influenza pathogenesis. The sex differences may be influenced by varying levels of steroid hormones, as immune cells like microglia express steroid hormone receptors and can be modulated by hormone levels. Steroid hormones, such as estradiol and testosterone, can regulate inflammatory responses through effects on transcriptional factors like NF-kB (Mckay and Cidlowski, 1999; Biswas et al., 2005; Chadwick et al., 2005), providing one mechanistic pathway for differential regulation of immunological homeostasis between sexes. Overall, our results demonstrate that primary hippocampal triple co-cultures serve as a potent model for investigating virus-induced effects on brain resident cells in a sex-specific manner.

Microglia play a pivotal role in the brain’s immune response. Our study delved into their sex-specific response to IAV infection. Microglia exhibit remarkable plasticity, allowing them to perform various functions in the CNS under both homeostatic and pathological conditions. They undergo morphological and functional transformations upon neuronal insult, migrate to lesion sites, and perform phagocytosis to clear debris and eliminate pathogens (Garden and Möller, 2006; Graeber, 2010). We observed a gradual increase in the percentage of hyper-ramified microglia following IAV infection in both sexes. Hyper-ramification of microglial processes has been previously observed in response to stimulation with the viral mimetic poly I:C (He et al., 2021) and is thought to play a role in synaptic modifications (Smith et al., 2019). Interestingly, only male-derived cultures exhibited a transformation to amoeboid microglia upon IAV infection. Amoeboid microglia, considered an ‘active’ state, have been previously noted upon infection, injury, and pathological processes. They play a key role during viral infection and in the healthy brain by eliminating debris and apoptotic cells (Stence et al., 2001; Helmut et al., 2011; Kettenmann et al., 2013).

Our findings indicate a sex-specific morphological response of microglia to IAV infection. To further explore functional changes, we analyzed the expression of ionized calcium-binding adapter molecule 1 (IBA1) and CD68, along with volumetric changes in lysosomes. IBA1 is involved in reorganization of the actin cytoskeleton by cross-linking actin to induce membrane ruffling (Sasaki et al., 2001) necessary for microglia motility and phagocytosis (Oshawa et al., 2000), thus linking it to microglia activity. In male-derived cultures, an increase in IBA1 expression suggested heightened activation due to IAV infection, whereas female-derived cultures showed a decrease. Additionally, CD68 expression increased in both hyper-ramified and amoeboid-shaped microglia in a sex-independent manner, indicating phagocytic activity and functional activation (Walker and Lue, 2015). The increased CD68 and decreased IBA1 expression in female-derived cultures indicates an altered microglial immunophenotype (Vidal-Itriago et al., 2022), pointing to an increase of a microglial subpopulation known as disease-associated microglia (DAM) (Hinwood et al., 2012). This finding is important for understanding the sex-specific mechanisms of neuroinflammation.

Considering the importance of microglia in synapse modification and circuit integrity, we analyzed changes in spine density upon infection in both sexes with and without microglial involvement. Interestingly, we observed an increase in spine density in female-derived cultures independent of the presence of microglia. IFN-β secretion might drive spinogenesis, as interferon signaling can increase astrocytic GLAST expression, suggesting modulation of glutamate uptake directly affects synaptic strength and spine density in an activity-dependent manner (Hosseini et al., 2020). An imbalance in glutamatergic synaptic transmission can lead to hyperexcitability and excitotoxicity (Düsedau et al., 2021). Spinogenesis may also be promoted through estradiol-mediated pathways especially in female-derived cultures (Jelks et al., 2007; Hojo et al., 2015; Sellers et al., 2015). Excitotoxicity might therefore be responsible for the spine loss observed 30 days after infection in females (Hosseini et al., 2018). In male-derived cultures, only a trend toward a higher spine density was observed, particularly in cultures without microglia. This, combined with increased pruning of postsynaptic structures by male microglia, suggests microglia may prune excessive spines, as reported by Chen et al. (2019). The observed decrease in mushroom-type spines and increase in thin spines in female neurons may represent a compensatory mechanism to counteract excessive excitability. We hypothesize that virus-induced phagocytosis of functional mushroom spines leads to increased thin spines and ultimately neurological impairments in female-derived cultures.

Microglial synaptic stripping can result from the engulfment of either presynaptic or postsynaptic terminals, representing different mechanisms to prevent neuropathological consequences like hyperexcitability or limit viral spread. During viral infection, complement component C3 localizes to presynaptic terminals triggering the expression of the C3a receptor on microglia and attracting them to neurons to engulf presynaptic compartments (Garber et al., 2019). This neuron-microglia interaction may represent a protective mechanism by removing presynapses, potentially preventing trans-synaptic viral spread and altered signaling from infected neurons, as observed after West Nile virus infection (Vasek et al., 2016). Our data show increased presynaptic stripping by microglia in both sexes, emphasizing the protective mechanism following neurotropic IAV infection. Remarkably, postsynaptic stripping was only observed in male cultures, suggesting a compensatory mechanism to avoid hyperexcitability. Hyper-ramified microglia, observed in both sexes, are hypothesized to be involved in presynaptic terminal engulfment to limit viral spread, while amoeboid microglia, observed specifically in male-derived cultures, may preferentially engulf postsynaptic terminals. This implies sex-specific microglial reactivity following neurotropic IAV infection, potentially leading to faster homeostatic recovery in males. This aligns with previous reports of higher susceptibility and more severe outcomes of IAV infection in females (Seki et al., 2018; vom Steeg and Klein, 2019; Wong et al., 2019; Humeniuk et al., 2023). In addition, the number of engulfed postsynaptic terminals was significantly lower in male-derived microglia than in female-derived microglia in control conditions, which may indicate sex-specific differences in the homeostatic functions of microglial cells that need to be clarified in the future investigations.

In conclusion, our study reveals a sex-specific microglial response to neurotropic IAV infection, with female-derived cultures showing greater interferon and pro-inflammatory chemokine secretion. Additionally, our data suggest sex-specific subpopulations of microglia differentially influenced by IAV infection. The preferential phagocytosis of postsynaptic compartments by amoeboid microglia in male-derived cultures may help prevent hyperexcitability. Investigating these intricate mechanisms promises insights into the pathogenesis of influenza-associated neurological disorders and the development of targeted therapeutic interventions.

## Data availability statement

The original contributions presented in this study are included in the article/Supplementary material, further inquiries can be directed to the corresponding author.

## Ethics statement

The animal study was approved by the local committees at TU Braunschweig and the authorities (LAVES, Oldenburg, Germany) according to the national guidelines of the animal welfare law in Germany (‘Tierschutzgesetz in der Fassung der Bekanntmachung vom 18. Mai 2006 (BGBl. I S. 1206, 1313). The study was conducted in accordance with the local legislation and institutional requirements.

## Author contributions

LG: Data curation, Formal analysis, Investigation, Methodology, Software, Writing – original draft. Writing – review and editing. IB: Data curation, Formal analysis, Investigation, Methodology, Writing – original draft, Writing – review and editing. NR: Methodology, Resources, Writing – original draft, Writing – review and editing. CS: Conceptualization, Methodology, Resources, Writing – original draft, Writing – review and editing. KM-P: Data curation, Formal analysis, Methodology, Software, Supervision, Validation, Writing – original draft, Writing – review and editing. SH: Conceptualization, Data curation, Formal analysis, Funding acquisition, Methodology, Software, Supervision, Validation, Writing – original draft, Writing – review and editing. MK: Conceptualization, Data curation, Formal analysis, Funding acquisition, Methodology, Software, Supervision, Validation, Writing – original draft, Writing – review and editing.

## References

[B1] Arganda-Carreras I. Fernández-González R. Muñoz-Barrutia A. Ortiz-De-Solorzano C. (2010). 3D reconstruction of histological sections: application to mammary gland tissue. *Microsc. Res. Tech*. 73, 1019–1029. 10.1002/jemt.20829 20232465

[B2] Bhattiprolu S. (2020). *python_for_microscopists. GitHub.* Available online at: https://github.com/bnsreenu/python_for_microscopists/blob/master/035-Cell%20Nuclei%20analysis%20using%20watershed.py (accessed May 27, 2024).

[B3] Biswas D. K. Singh S. Shi Q. Paradee A. B. Iglehart D. J. (2005). Crossroads of estrogen receptor and NF-κB signaling. *Sci. STKE* 2005:e27. 10.1126/stke.2882005pe27 15956359

[B4] Bohmwald K. Andrade C. A. Kalergis A. M. (2021). Contribution of pro-inflammatory molecules induced by respiratory virus infections to neurological disorders. *Pharmaceuticals* 14:340. 10.3390/ph14040340 33917837 PMC8068239

[B5] Bourne J. Harris K. M. (2007). Do thin spines learn to be mushroom spines that remember? *Curr. Opin. Neurobiol.* 17 381–386. 10.1016/j.conb.2007.04.009 17498943

[B6] Campos A. B. Duarte-Silva S. Ambrósio A. F. Maciel P. Fernandes B. (2021). MorphData: Automating the data extraction process of morphological features of microglial cells in ImageJ. *bioRxiv* [Preprint]. 10.1101/2021.08.05.455282

[B7] Chadwick C. C. Chippari S. Matelan E. Borges-Marcucci L. Eckert A. M. Keith J. C. (2005). Identification of pathway-selective estrogen receptor ligands that inhibit NF-B transcriptional activity. *Proc. Natl. Acad. Sci. U.S.A.* 15:102. 10.1073/pnas.0405841102 PMC548967 15699342

[B8] Chen Z. Zhong D. Li G. (2019). The role of microglia in viral encephalitis: A review. *J. Neuroinflamm.* 16:76. 10.1186/s12974-019-1443-2 30967139 PMC6454758

[B9] Cocoros N. M. Svensson E. Komjáthine Szépligeti S. Viborg Vestergaard S. Szentkúti P. Thomsen R. W. (2021). Long-term risk of Parkinson disease following influenza and other infections. *JAMA Neurol.* 78 1–11. 34694344 10.1001/jamaneurol.2021.3895 PMC8546623

[B10] Crighton E. J. Elliott S. J. Kanaroglou P. Moineddin R. Upshur R. E. G. (2008). Spatio-temporal analysis of pneumonia and influenza hospitalizations in Ontario, Canada. *Geospat. Health* 2 191–202. 10.4081/gh.2008.243 18686268

[B11] Crighton E. J. Moineddin R. Mamdani M. Upshur R. E. G. (2004). Influenza and pneumonia hospitalizations in Ontario: A time-series analysis. *Epidemiol. Infect.* 132 1167–1174. 10.1017/S0950268804002924 15635976 PMC2870210

[B12] de Araujo M. E. G. Liebscher G. Hess M. W. Huber L. A. (2020). Lysosomal size matters. *Traffic* 21 60–75. 10.1111/tra.12714 31808235 PMC6972631

[B13] DiSabato D. J. Quan N. Godbout J. P. (2016). Neuroinflammation: The devil is in the details. *J. Neurochem.* 139 136–153. 10.1111/jnc.13607 26990767 PMC5025335

[B14] Düsedau H. P. Steffen J. Figueiredo C. A. Boehme J. D. Schultz K. Erck C. (2021). Influenza A virus (H1N1) infection induces microglial activation and temporal dysbalance in glutamatergic synaptic transmission. *mBio* 12:e0177621. 10.1128/mBio.01776-21 34700379 PMC8546584

[B15] Edler M. K. Mhatre-Winters I. Richardson J. R. (2021). Microglia in aging and Alzheimer’s disease: A comparative species review. *Cells* 10:1138. 10.3390/cells10051138 34066847 PMC8150617

[B16] Fernández-Arjona M. D. M. Grondona J. M. Granados-Durán P. Fernández-Llebrez P. López-Ávalos M. D. (2017). Microglia morphological categorization in a rat model of Neuroinflammation by hierarchical cluster and principal components analysis. *Front. Cell. Neurosci*. 11:235. 10.3389/fncel.2017.00235 28848398 PMC5550745

[B17] Frank-Cannon T. C. Alto L. T. McAlpine F. E. Tansey M. G. (2009). Does neuroinflammation fan the flame in neurodegenerative diseases? *Mol. Neurodegener.* 4:47. 10.1186/1750-1326-4-47 19917131 PMC2784760

[B18] Gabriel G. Dauber B. Wolff T. Planz O. Klenk H.-D. Stech J. (2005). The viral polymerase mediates adaptation of an avian influenza virus to a mammalian host. *Proc. Natl. Acad. Sci. U.S.A.* 102 18590–18595. 16339318 10.1073/pnas.0507415102 PMC1317936

[B19] Galic M. A. Riazi K. Pittman Q. J. (2012). Cytokines and brain excitability. *Front. Neuroendocrinol.* 33:116–125. 10.1016/j.yfrne.2011.12.002 22214786 PMC3547977

[B20] Garber C. Soung A. Vollmer L. L. Kanmogne M. Last A. Brown J. (2019). T cells promote microglia-mediated synaptic elimination and cognitive dysfunction during recovery from neuropathogenic flaviviruses. *Nat. Neurosci.* 22 1276–1288. 10.1038/s41593-019-0427-y 31235930 PMC6822175

[B21] Garden G. A. Möller T. (2006). Microglia biology in health and disease. *J. Neuroimmune Pharmacol.* 1 127–137. 10.1007/s11481-006-9015-5 18040779

[B22] Graeber M. B. (2010). Changing face of microglia. *Science* 330 783–788. 21051630 10.1126/science.1190929

[B23] Guneykaya D. Ivanov A. Hernandez D. P. Haage V. Wojtas B. Meyer N. (2018). Transcriptional and translational differences of microglia from male and female brains. *Cell Rep.* 24:2773–2783.e6. 10.1016/j.celrep.2018.08.001 30184509

[B24] Guyon A. Banisadr G. Rovère C. Cervantes A. Kitabgi P. Melik-Parsadaniantz S. (2005). Complex effects of stromal cell-derived factor-1α on melanin-concentrating hormone neuron excitability. *Eur. J. Neurosci.* 21 701–710. 10.1111/j.1460-9568.2005.03890.x 15733088

[B25] Han J. Fan Y. Zhou K. Blomgren K. Harris R. A. (2021). Uncovering sex differences of rodent microglia. *J. Neuroinflamm.* 18:74. 10.1186/s12974-021-02124-z 33731174 PMC7972194

[B26] He Y. Taylor N. Yao X. Bhattacharya A. (2021). Mouse primary microglia respond differently to LPS and poly(I:C) in vitro. *Sci. Rep.* 11:10447. 10.1038/s41598-021-89777-1 34001933 PMC8129154

[B27] Heinisch S. Kirby L. G. (2010). SDF-1α/CXCL12 enhances GABA and glutamate synaptic activity at serotonin neurons in the rat dorsal raphe nucleus. *Neuropharmacology* 58 501–514. 10.1016/j.neuropharm.2009.08.022 19755127 PMC2813394

[B28] Helmut K. Hanisch U. K. Noda M. Verkhratsky A. (2011). Physiology of microglia. *Physiol. Rev.* 91 461–553. 10.1152/physrev.00011.2010 21527731

[B29] Heneka M. T. Kummer M. P. Latz E. (2014). Innate immune activation in neurodegenerative disease. *Nat. Rev. Immunol.* 14 463–477. 10.1038/nri3705 24962261

[B30] Hinwood M. Morandini J. Day T. A. Walker F. R. (2012). Evidence that microglia mediate the neurobiological effects of chronic psychological stress on the medial prefrontal cortex. *Cereb. Cortex* 22 1442–1454. 10.1093/cercor/bhr229 21878486

[B31] Hojo Y. Munetomo A. Mukai H. Ikeda M. Sato R. Hatanaka Y. (2015). Estradiol rapidly modulates spinogenesis in hippocampal dentate gyrus: Involvement of kinase networks. *Horm. Behav.* 74 149–156. 10.1016/j.yhbeh.2015.06.008 26122288

[B32] Hosking M. P. Lane T. E. (2010). The role of chemokines during viral infection of the CNS. *PLoS Pathog.* 6:e1000937. 10.1371/journal.ppat PMC2912390 20686655

[B33] Hosseini S. Michaelsen-Preusse K. Grigoryan G. Chhatbar C. Kalinke U. Korte M. (2020). Type I interferon receptor signaling in astrocytes regulates hippocampal synaptic plasticity and cognitive function of the healthy CNS. *Cell Rep.* 31:107666. 10.1016/j.celrep.2020.107666 32433975

[B34] Hosseini S. Wilk E. Michaelsen-Preusse K. Gerhauser I. Baumgärtner W. Geffers R. (2018). Long-term neuroinflammation induced by influenza a virus infection and the impact on hippocampal neuron morphology and function. *J. Neurosci.* 38 3060–3080. 10.1523/JNEUROSCI.1740-17.2018 29487124 PMC6596076

[B35] Humeniuk P. Barrett A. Axelsson H. Corciulo C. Drevinge C. Pons A. D. C. (2023). Profiling of innate and adaptive immune cells during influenza virus infection reveals sex bias in invariant natural killer T (iNKT) cells. *Immun. Inflamm. Dis.* 11:e837. 10.1002/iid3.837 37102646 PMC10091374

[B36] Jang H. Boltz D. McClaren J. Pani A. K. Smeyne M. Korff A. (2012). Inflammatory effects of highly pathogenic H5N1 influenza virus infection in the CNS of mice. *J. Neurosci.* 32 1545–1559. 10.1523/JNEUROSCI.5123-11.2012 22302798 PMC3307392

[B37] Jang H. Boltz D. Sturm-Ramirez K. Shepherd K. R. Jiao Y. Webster R. (2009). Highly pathogenic H5N1 influenza virus can enter the central nervous system and induce neuroinflammation and neurodegeneration. *Proc. Natl. Acad. Sci. U.S.A.* 106 14063–14068. 19667183 10.1073/pnas.0900096106 PMC2729020

[B38] Jelks K. B. Wylie R. Floyd C. L. McAllister A. K. Wise P. (2007). Estradiol targets synaptic proteins to induce glutamatergic synapse formation in cultured hippocampal neurons: Critical role of estrogen receptor-α. *J. Neurosci.* 27 6903–6913. 10.1523/JNEUROSCI.0909-07.2007 17596438 PMC6672227

[B39] Joynt P. (2018). Acute myocardial infarction after laboratory-confirmed influenza infection. *J. Emerg. Med.* 54 581–582. 10.1016/j.jemermed.2018.02.026

[B40] Jurgens H. A. Amancherla K. Johnson R. W. (2012). Influenza infection induces neuroinflammation, alters hippocampal neuron morphology, and impairs cognition in adult mice. *J. Neurosci.* 32 3958–3968. 10.1523/JNEUROSCI.6389-11.2012 22442063 PMC3353809

[B41] Karperien A. L. (2007-2012). *Fraclac for ImageJ*. Albury-Wodonga: Charles Sturt University, 1–36. Available online at: https://imagej.net/ij/plugins/fraclac/FLHelp/Introduction.htm

[B42] Kettenmann H. Kirchhoff F. Verkhratsky A. (2013). Microglia: New roles for the synaptic stripper. *Neuron* 77 10–18. 10.1016/j.neuron.2012.12.023 23312512

[B43] Klein S. L. Roberts C. W. (2010). *Sex hormones and immunity to infection.* Springer-Verlag.

[B44] Larcombe A. N. Foong R. E. Bozanich E. M. Berry L. J. Garratt L. W. Gualano R. C. (2011). Sexual dimorphism in lung function responses to acute influenza A infection. *Influenza Other Respir Viruses* 5 334–342. 10.1111/j.1750-2659.2011.00236.x 21668688 PMC4942045

[B45] Levet F. Tønnesen J. Nägerl U. V. Sibarita J. B. (2020). SpineJ: A software tool for quantitative analysis of nanoscale spine morphology. *Methods* 174 49–55. 10.1016/j.ymeth.2020.01.020 32006677

[B46] Li L. Mao S. Wang J. Ding X. Zen J. Y. (2019). Viral infection and neurological disorders—potential role of extracellular nucleotides in neuroinflammation. *ExRNA* 1:26. 10.1186/s41544-019-0031-z

[B47] Lima M. N. Barbosa-Silva M. C. Maron-Gutierrez T. (2022). Microglial priming in infections and its risk to neurodegenerative diseases. *Front. Cell. Neurosci.* 16:878987. 10.3389/fncel.2022.878987 35783096 PMC9240317

[B48] Lynch M. A. (2022). Exploring sex-related differences in microglia may be a game-changer in precision medicine. *Front. Aging Neurosci.* 14:868448. 10.3389/fnagi.2022.868448 35431903 PMC9009390

[B49] Mckay L. I. Cidlowski J. A. (1999). Molecular control of immune/inflammatory responses: Interactions between nuclear factor-b and steroid receptor-signaling pathways. *Endocr. Rev.* 20 435–459. 10453354 10.1210/edrv.20.4.0375

[B50] Michaelsen K. Zagrebelsky M. Berndt-Huch J. Polack M. Buschler A. Sendtner M. (2010). Neurotrophin receptors TrkB.T1 and p75NTR cooperate in modulating both functional and structural plasticity in mature hippocampal neurons. *Eur. J. Neurosci.* 32 1854–1865. 10.1111/j.1460-9568.2010.07460.x 20955473

[B51] Michell-Robinson M. A. Touil H. Healy L. M. Owen D. R. Durafourt B. A. Bar-Or A. (2015). Roles of microglia in brain development, tissue maintenance and repair. *Brain* 138 1138–1159. 10.1093/brain/awv066 25823474 PMC5963417

[B52] Morgan R. Klein S. L. (2019). The intersection of sex and gender in the treatment of influenza. *Curr. Opin. Virol.* 35 35–41. 10.1016/j.coviro.2019.02.009 30901632 PMC6556398

[B53] Muñoz-Moreno R. Martínez-Romero C. García-Sastre A. (2021). Induction and evasion of type-i interferon responses during influenza a virus infection. *Cold Spring Harb. Perspect. Med.* 11:a038414. 10.1101/cshperspect.a038414 32661015 PMC8485741

[B54] Murdaugh L. B. Mendoza-Romero H. N. Fish E. W. Parnell S. E. (2018). A novel method for determining sex in late term gestational mice based on the external genitalia. *PLoS One* 13:e0194767. 10.1371/journal.pone.0194767 29617407 PMC5884523

[B55] Nakamura Y. (2002). Regulating factors for microglial activation. *Biol. Pharm. Bull.* 25 945–953. 12186424 10.1248/bpb.25.945

[B56] Nissen J. C. (2017). Microglial function across the spectrum of age and gender. *Int. J. Mol. Sci.* 18:561. 10.3390/ijms18030561 28273860 PMC5372577

[B57] Oshawa K. Imai Y. Kanazawa H. Sasaki Y. Kohsaka S. (2000). Involvement of Iba1 in membrane ruffling and phagocytosis of macrophages/microglia. *J. Cell Sci.* 113 3073–3084. 10934045 10.1242/jcs.113.17.3073

[B58] Parkhurst C. N. Yang G. Ninan I. Savas J. N. Yates J. R. Lafaille J. J. (2013). Microglia promote learning-dependent synapse formation through brain-derived neurotrophic factor. *Cell* 155 1596–1609. 10.1016/j.cell.2013.11.030 24360280 PMC4033691

[B59] Perlmutter L. Scott S. Barron E. Chui H. (1992). MHC Class 11-positive microglia in human brain: Association with Alzheimer lesions. *J. Neurosci. Res.* 33 549–558. 1484388 10.1002/jnr.490330407

[B60] Peters A. Kaisermann-Abramof R. (1970). The small pyramidal neuron of the rat cerebral cortex. The perikaryon, dendrites and spines. *Am. J. Anat.* 127 321–355. 4985058 10.1002/aja.1001270402

[B61] Pujantell M. Altfeld M. (2022). Consequences of sex differences in type I IFN responses for the regulation of antiviral immunity. *Front. Immunol.* 13:986840. 10.3389/fimmu.2022.986840 36189206 PMC9522975

[B62] Quach C. Piché-Walker L. Platt R. Moore D. (2003). Risk factors associated with severe influenza infections in childhood: Implication for vaccine strategy. *Pediatrics* 112 e197–e201. 12949312 10.1542/peds.112.3.e197

[B63] Ramesh G. Maclean A. G. Philipp M. T. (2013). Cytokines and chemokines at the crossroads of neuroinflammation, neurodegeneration, and neuropathic pain. *Mediat. Inflamm.* 2013:480739. 10.1155/2013/480739 23997430 PMC3753746

[B64] Sahu P. S. Ter E. (2018). Interactions between neurotropic pathogens, neuroinflammatory pathways, and autophagic neural cell death. *Neuroimmunol. Neuroinflamm.* 5:2. 10.20517/2347-8659.2017.43

[B65] Sasaki Y. Ohsawa K. Kanazawa H. Kohsaka S. Imai Y. (2001). Iba1 is an actin-cross-linking protein in macrophages/microglia. *Biochem. Biophys. Res. Commun.* 286 292–297. 10.1006/bbrc.2001.5388 11500035

[B66] Schindelin J. Arganda-Carreras I. Frise E. Kaynig V. Longair M. Pietzsch T. (2012). Fiji: An open-source platform for biological-image analysis. *Nat. Methods* 9 676–682. 10.1038/nmeth.2019 22743772 PMC3855844

[B67] Seki Y. Onose A. Murayama T. Koide C. Sugaya N. (2018). Influenza vaccine showed a good preventive effect against influenza-associated hospitalization among elderly patients, during the 2016/17 season in Japan. *J. Infect. Chemother.* 24 873–880. 10.1016/j.jiac.2018.07.013 30100400

[B68] Sellers K. J. Erli F. Raval P. Watson I. A. Chen D. Srivastava D. P. (2015). Rapid modulation of synaptogenesis and spinogenesis by 17β-estradiol in primary cortical neurons. *Front. Cell. Neurosci.* 9:137. 10.3389/fncel.2015.00137 25926772 PMC4396386

[B69] Sellers S. A. Hagan R. S. Hayden F. G. Fischer W. A. (2017). The hidden burden of influenza: A review of the extra-pulmonary complications of influenza infection. *Influenza Other Respir Viruses* 11 372–393. 10.1111/irv.12470 28745014 PMC5596521

[B70] Shim J. M. Kim J. Tenson T. Min J. Y. Kainov D. E. (2017). Influenza virus infection, interferon response, viral counter-response, and apoptosis. *Viruses* 9:223. 10.3390/v9080223 28805681 PMC5580480

[B71] Smith K. L. Kassem M. S. Clarke D. J. Kuligowski M. P. Bedoya-Pérez M. A. Todd S. M. (2019). Microglial cell hyper-ramification and neuronal dendritic spine loss in the hippocampus and medial prefrontal cortex in a mouse model of PTSD. *Brain Behav. Immun.* 80 889–899. 10.1016/j.bbi.2019.05.042 31158497

[B72] Stence N. Waite M. Dailey M. (2001). Dynamics of microglial activation: A confocal time-lapse analysis in hippocampal slices. *Gila* 33 66–256. 11241743

[B73] Streit W. J. Mrak R. E. Griffin W. S. T. (2004). Microglia and neuroinflammation: A pathological perspective. *J. Neuroinflamm.* 1:14. 10.1186/1742-2094-1-14 15285801 PMC509427

[B74] Toovey S. Jick S. S. Meier C. R. (2011). Parkinson’s disease or Parkinson symptoms following seasonal influenza. *Influenza Other Respir Viruses* 5 328–333. 10.1111/j.1750-2659.2011.00232.x 21668692 PMC4942044

[B75] Vasek M. J. Garber C. Dorsey D. Durrant D. M. Bollman B. Soung A. (2016). A complement-microglial axis drives synapse loss during virus-induced memory impairment. *Nature* 534 538–543. 10.1038/nature18283 27337340 PMC5452615

[B76] Vidal-Itriago A. Radford R. A. W. Aramideh J. A. Maurel C. Scherer N. M. Don E. K. (2022). Microglia morphophysiological diversity and its implications for the CNS. *Front. Immunol.* 13:997786. 10.3389/fimmu.2022.997786 36341385 PMC9627549

[B77] vom Steeg L. G. Klein S. L. (2019). Sex and sex steroids impact influenza pathogenesis across the life course. *Semin. Immunopathol.* 41 189–194. 10.1007/s00281-018-0718-5 30298431 PMC6370518

[B78] Vom Steeg L. G. Vermillion M. S. Hall O. J. Alam O. McFarland R. Chen H. (2016). Age and testosterone mediate influenza pathogenesis in male mice. *Am. J. Physiol. Lung Cell Mol. Physiol.* 311 L1234–L1244. 10.1152/ajplung.00352.2016 27815260 PMC5206399

[B79] Wake H. Moorhouse A. J. Jinno S. Kohsaka S. Nabekura J. (2009). Resting microglia directly monitor the functional state of synapses in vivo and determine the fate of ischemic terminals. *J. Neurosci.* 29 3974–3980. 10.1523/JNEUROSCI.4363-08.2009 19339593 PMC6665392

[B80] Walker D. G. Lue L. F. (2015). Immune phenotypes of microglia in human neurodegenerative disease: Challenges to detecting microglial polarization in human brains. *Alzheimers Res. Ther.* 7:56. 10.1186/s13195-015-0139-9 26286145 PMC4543480

[B81] Wolterink-Donselaar I. G. (2009). A method for gender determination in newborn dark pigmented mice. *Lab. Anim.* 38 35–38. 10.1038/laban0109-35 19112448

[B82] Wong K. C. Luscombe G. M. Hawke C. (2019). Influenza infections in Australia 2009-2015: Is there a combined effect of age and sex on susceptibility to virus subtypes? *BMC Infect. Dis.* 19:42. 10.1186/s12879-019-3681-4 30630435 PMC6327581

[B83] Yanguas-Casás N. (2020). Physiological sex differences in microglia and their relevance in neurological disorders. *Neuroimmunol. Neuroinflamm.* 7 13–22. 10.20517/2347-8659.2019.31

[B84] Yanguas-Casás N. Crespo-Castrillo A. de Ceballos M. L. Chowen J. A. Azcoitia I. Arevalo M. A. (2018). Sex differences in the phagocytic and migratory activity of microglia and their impairment by palmitic acid. *Glia* 66 522–537. 10.1002/glia.23263 29139169

[B85] Yuste R. (2013). Electrical compartmentalization in dendritic spines. *Annu. Rev. Neurosci.* 36 429–449. 23724997 10.1146/annurev-neuro-062111-150455

[B86] Zhou L. Miranda-Saksena M. Saksena N. K. (2013). Viruses and neurodegeneration. *Virol. J.* 10:172. 10.1186/1743-422X-10-172 23724961 PMC3679988

