# Supplementary material for: H7N7 viral infection elicits pronounced, sex-specific neuroinflammatory responses in vitro
Source: Front Cell Neurosci. 2024 Aug 7;18:1444876. doi: 10.3389/fncel.2024.1444876 (PMC11335524; doi:10.3389/fncel.2024.1444876)
Supplement: Supplementary file 1 [file Data_Sheet_1.PDF]

Supplementary Material

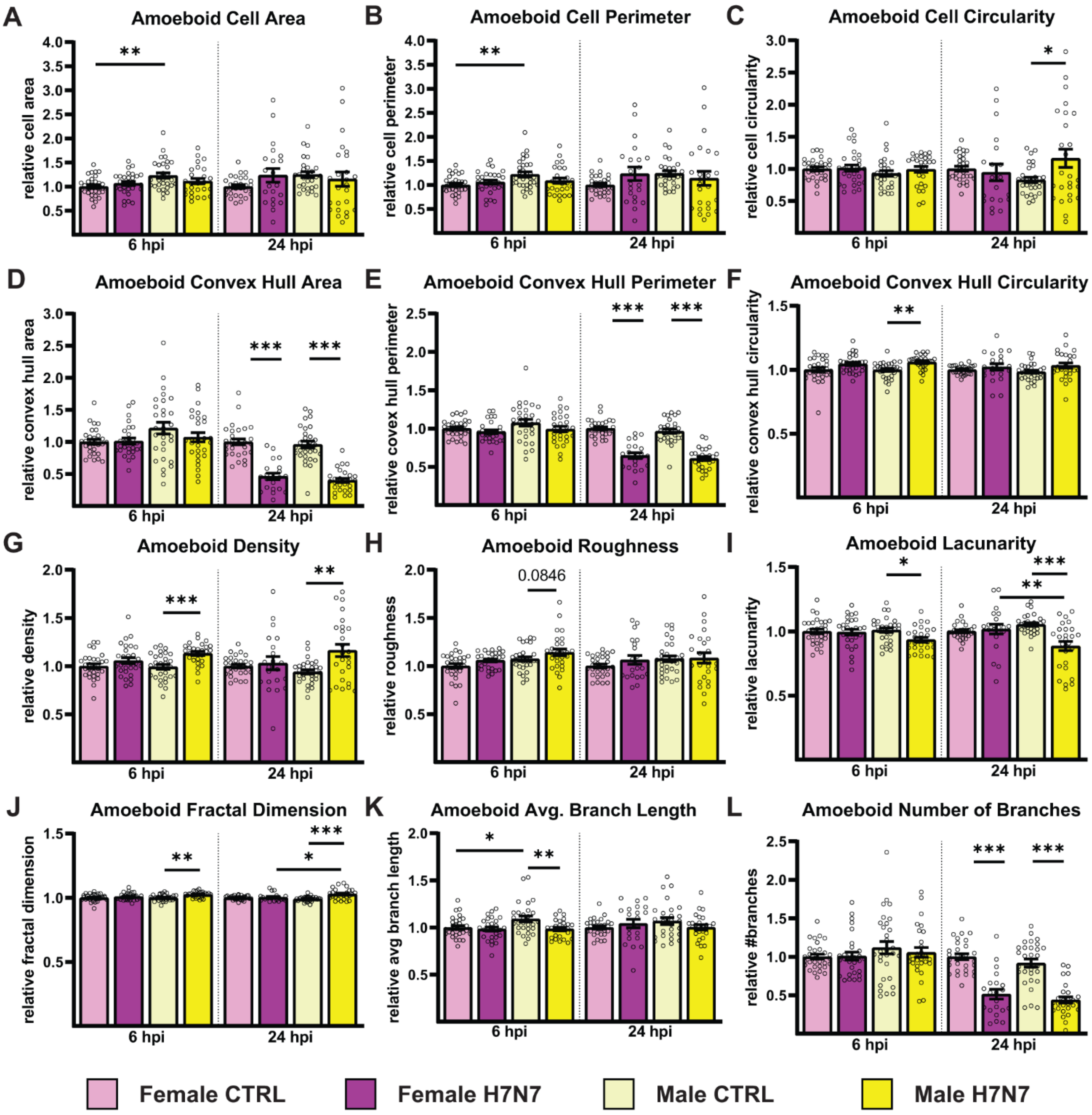

Supplementary Figure 1. Morphological characteristics of amoeboid-shaped microglia in female-and male-derived primary cultures after infection with influenza A/Seal/Mass/1/80 rSC35M (H7N7) virus. The following morphological characteristics were investigated: **(A)** cell area, **(B)** cell perimeter, **(C)** cell circularity, **(D)** convex hull area, **(E)** convex hull perimeter, **(F)** convex hull circularity, **(G)** density, **(H)** roughness, **(I)** lacunarity, **(J)** fractal dimension, **(K)** average branch length, **(L)** number of branches. Number of experiments, N = 3, n = 10 images per group and cell preparation round. Data are

presented as mean ± SEM and were analyzed by two-way ANOVA followed by post hoc Tukey test. *p < 0.05, **p < 0.01 and ***p < 0.001.

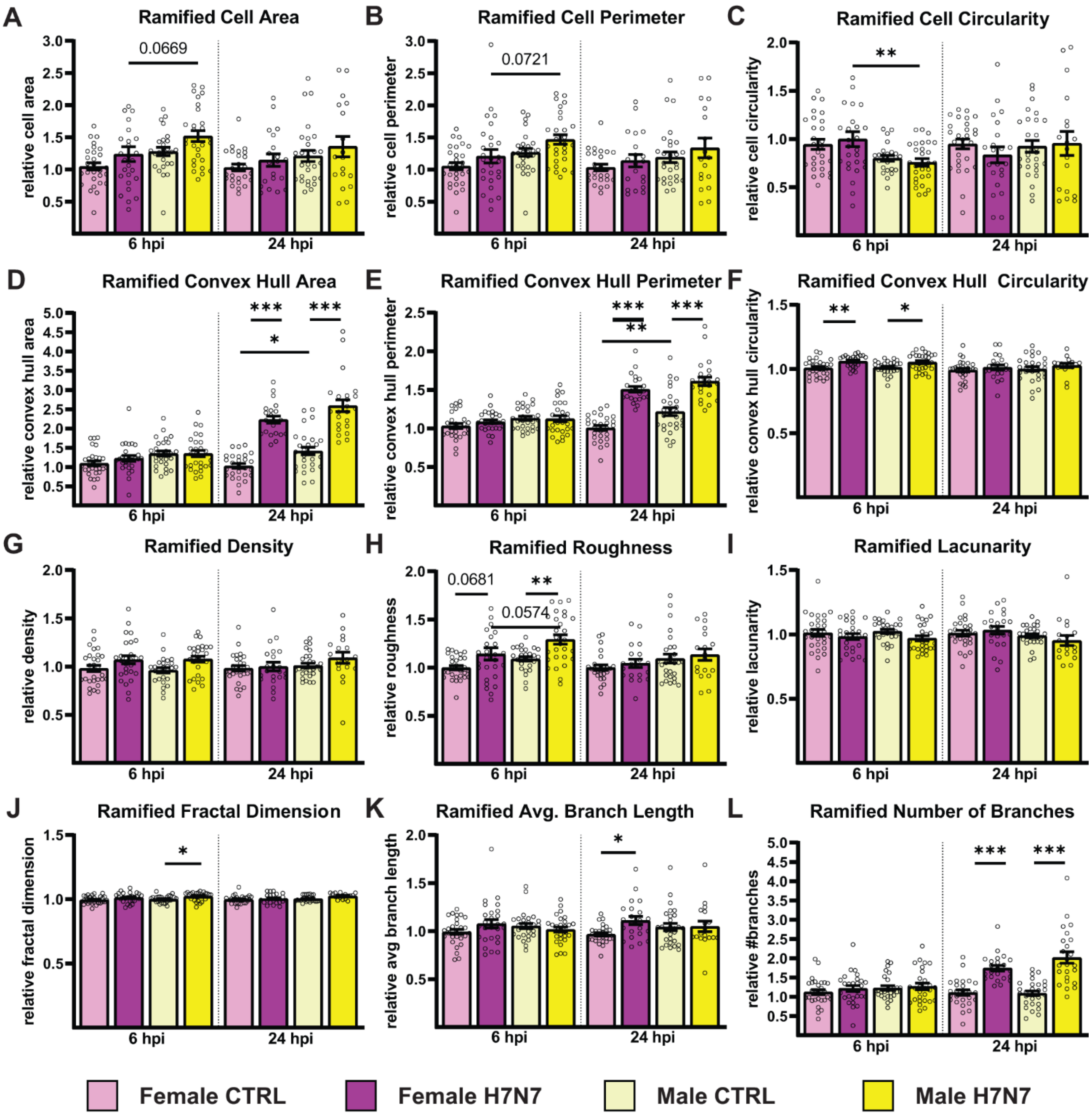

Supplementary Figure 2. Morphological characteristics of (hyper-)ramified microglia in female-and male-derived primary cultures after infection with influenza A/Seal/Mass/1/80 rSC35M (H7N7) virus. The following morphological characteristics were investigated: **(A)** cell area, **(B)** cell perimeter, **(C)** cell circularity, **(D)** convex hull area, **(E)** convex hull perimeter, **(F)** convex hull circularity, **(G)** density, **(H)** roughness, **(I)** lacunarity, **(J)** fractal dimension, **(K)** average branch length, **(L)** number of

branches. Number of experiments, $N = 3$, $n = 10$ images per group and cell preparation round. Data are presented as mean ± SEM and were analyzed by two-way ANOVA followed by post hoc Tukey test. *$p < 0.05$, **$p < 0.01$ and ***$p < 0.001$.

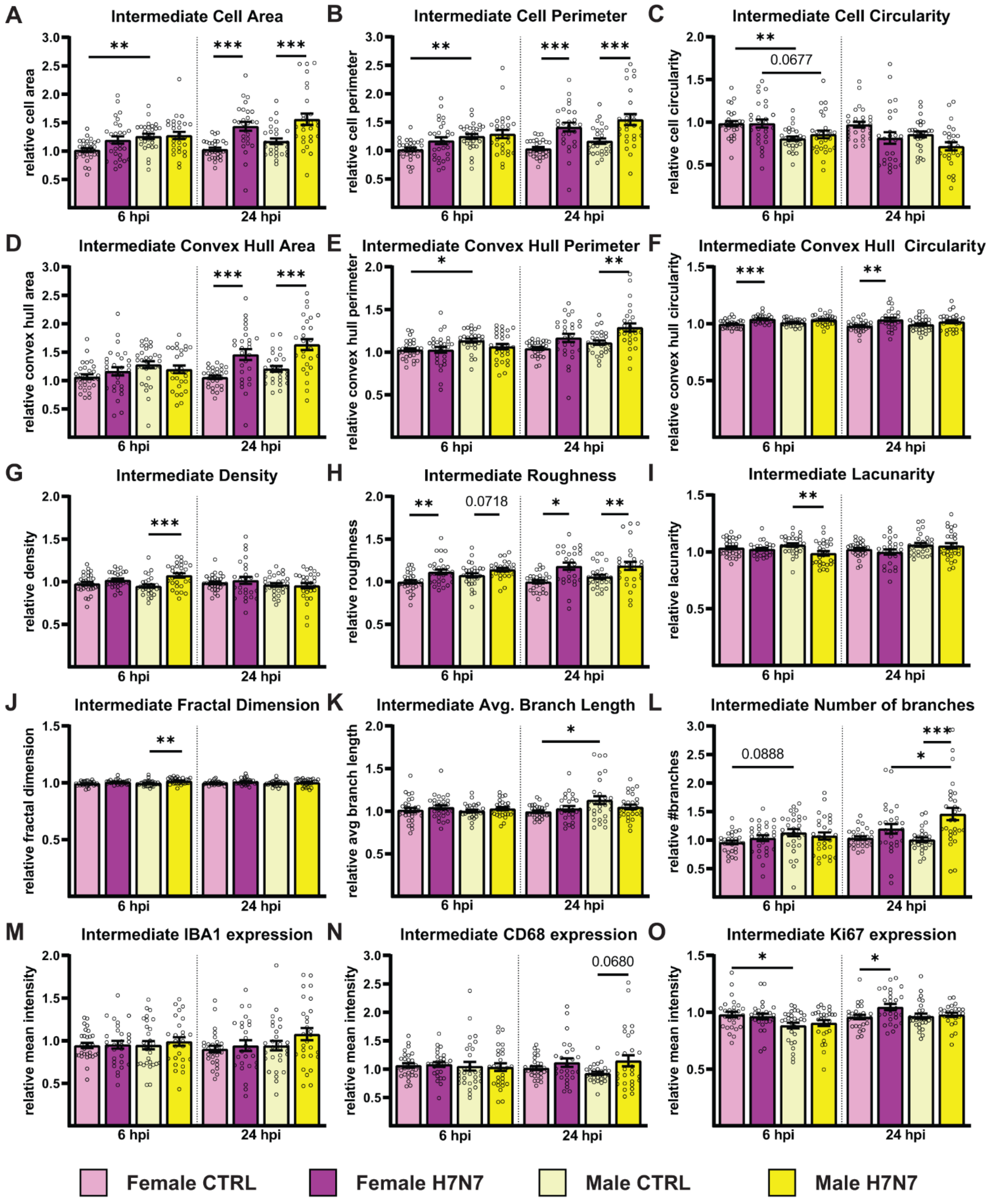

Supplementary Figure 3. Morphological characteristics of intermediate microglia in female-and male-derived primary cultures after infection with influenza A/Seal/Mass/1/80 rSC35M (H7N7) virus. The following morphological characteristics were investigated: **(A)** cell area, **(B)** cell perimeter, **(C)**

cell circularity, **(D)** convex hull area, **(E)** convex hull perimeter, **(F)** convex hull circularity, **(G)** density, **(H)** roughness, **(I)** lacunarity, **(J)** fractal dimension, **(K)** average branch length, **(L)** number of branches. Number of experiments, N = 3, n = 10 images per group and cell preparation round. Data are presented as mean ± SEM and were analyzed by two-way ANOVA followed by post hoc Tukey test. *p < 0.05, **p < 0.01 and ***p < 0.001.

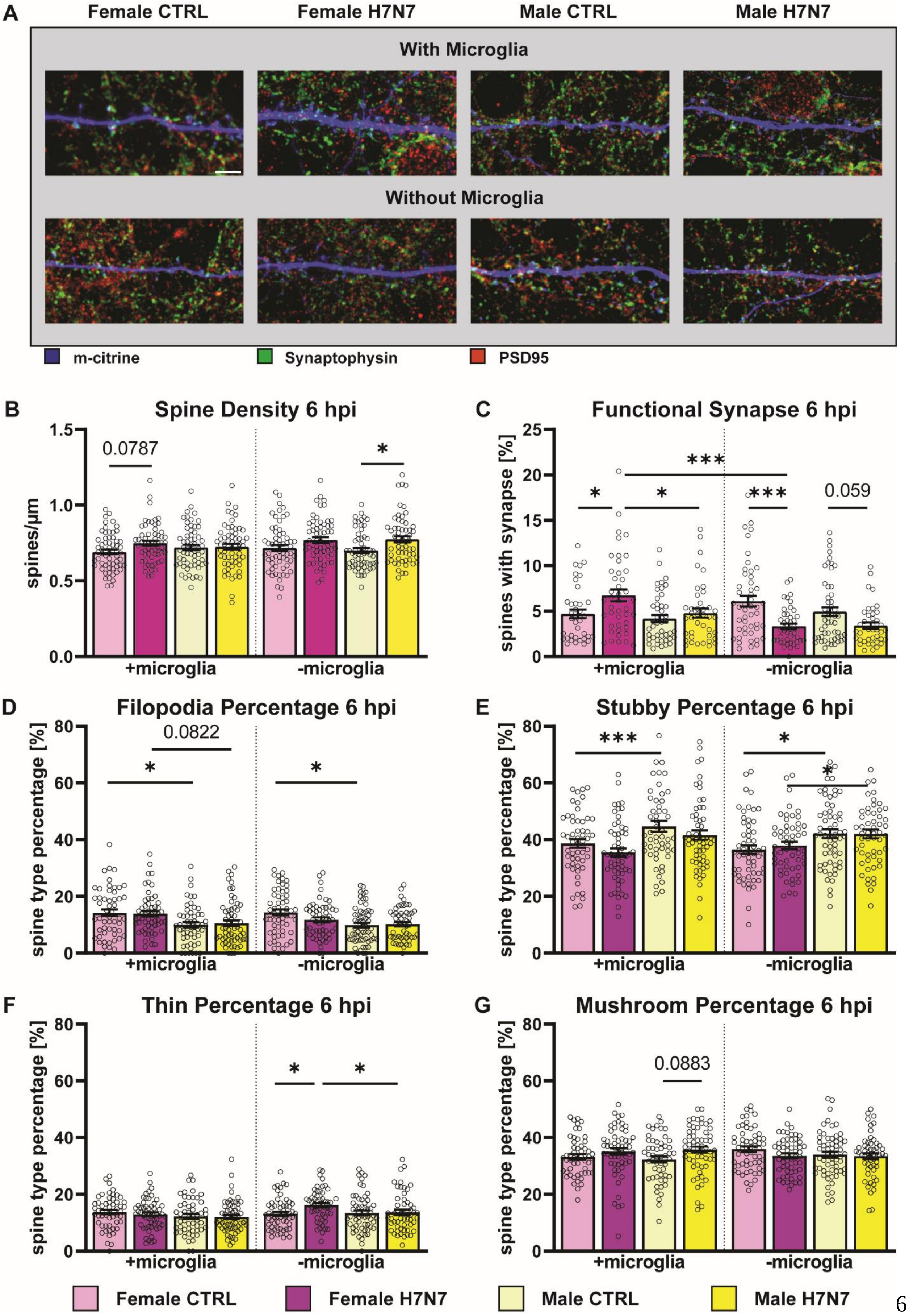
A
Female CTRL
Female H7N7
Male CTRL
Male H7N7
With Microglia
Without Microglia
m-citrine
Synaptophysin
PSD95
B
Spine Density 6 hpi
spines/µm
0.0787
*
+microglia
-microglia
C
Functional Synapse 6 hpi
spines with synapse [%]
***
*
*
***
0.059
+microglia
-microglia
D
Filopodia Percentage 6 hpi
spine type percentage [%]
0.0822
*
*
+microglia
-microglia
E
Stubby Percentage 6 hpi
spine type percentage [%]
***
*
*
+microglia
-microglia
F
Thin Percentage 6 hpi
spine type percentage [%]
*
*
+microglia
-microglia
G
Mushroom Percentage 6 hpi
spine type percentage [%]
0.0883
+microglia
-microglia
Female CTRL
Female H7N7
Male CTRL
Male H7N7

Supplementary Figure 4. Numerical and morphological changes in dendritic spine as well as changes in number of functional synapses upon viral infection can lead to detrimental neurological consequences. **(A)** Example images displaying dendritic spines (blue), presynaptic (green) and postsynaptic terminals (red). Scale bar: 5 µm. **(B)** Number of dendritic spines increased in female-derived cultures with microglia involvement and without microglia involvement in male-derived cultures at 6 hpi. **(C)** Number of functional synapses increased in female-derived cultures in a microglia-dependent manner and decreased without microglia presence. In male-derived cultures, a decrease in functional synapses was seen in microglia absence. Morphology of dendritic spines can indicate their functional status. **(D)** A higher filopodia type frequency was seen in female-derived cultures compared to male-derived cultures independent of microglia presence. **(E)** A lower stubby spine percentage was observed in female-derived cultures compared to male-derived cultures independent of microglia presence. **(F)** An increase in the thin spine percentage was only seen in female-derived cultures without microglia involvement 6 hpi. **(G)** Proportion of mushroom type spines increased only in male-derived cultures with microglia presence at 6 hpi. Number of experiments, N = 3, n = 20 images per group and cell preparation round. Data are presented as mean ± SEM and were analyzed by two-way ANOVA followed by post hoc Tukey test. *$p < 0.05$ and ***$p < 0.001$.

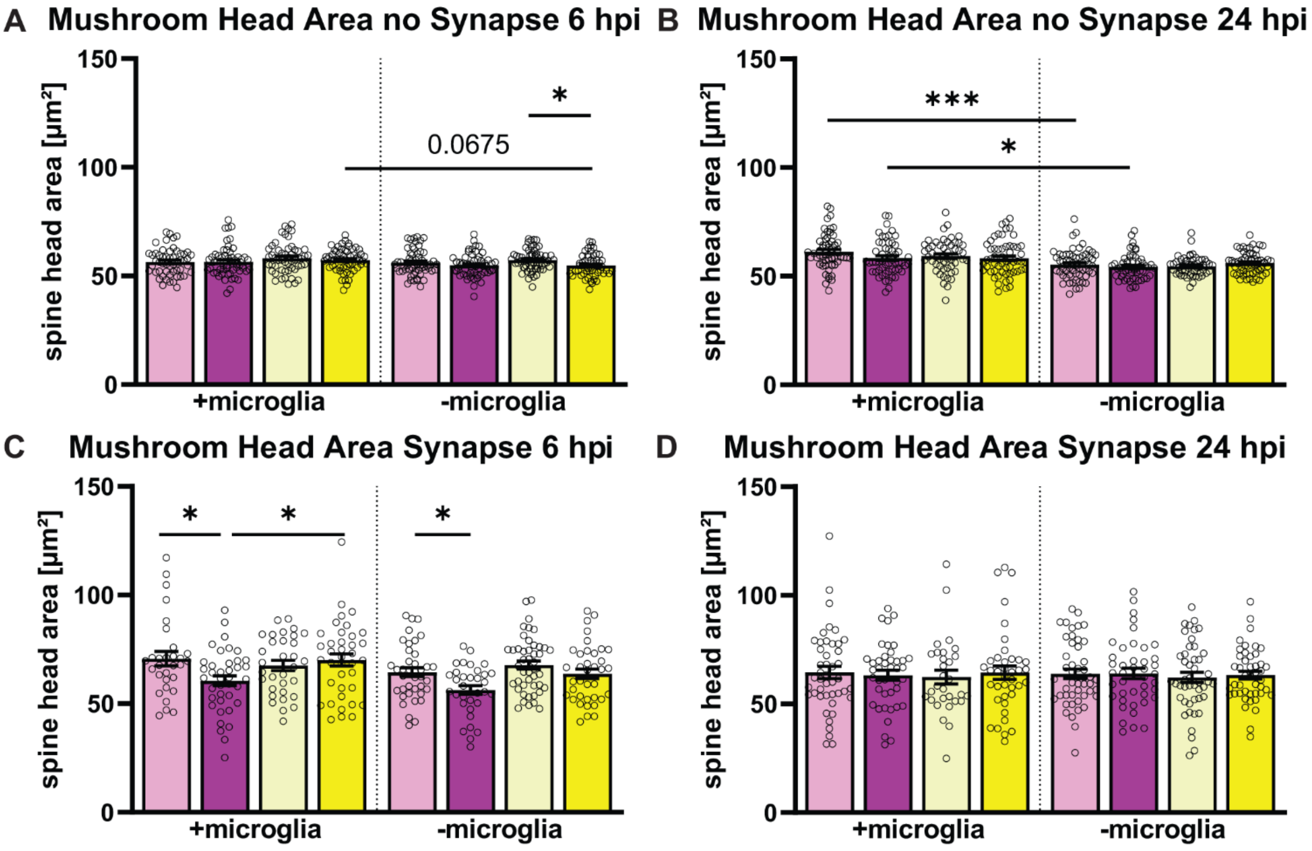

Supplementary Figure 5. Dendritic spine head areas can indicate the functionality and strength of the according spine as size and shape of spines can be correlated to the abundance of receptors within the cellular membrane of the PSD. **(A)** Mushroom-type spine head area without a synapse decreased only in male-derived cultures without microglia presence. **(B)** At 24 hpi, mushroom spines without a

synapse showed a greater spine head area with microglia involvement in both sexes. **(C)** Spine head areas of mushroom type spines with a synapse decreased in female-derived cultures independent of microglia presence at 6 hpi, whereby no change was seen in male-derived cultures. **(D)** At 24 hpi, no differences in mushroom spine head areas were observed. Number of experiments, N = 3, n = 20 images per group and cell preparation round. Data are presented as mean ± SEM and were analyzed by two-way ANOVA followed by post hoc Tukey test. *p < 0.05 and ***p < 0.001.

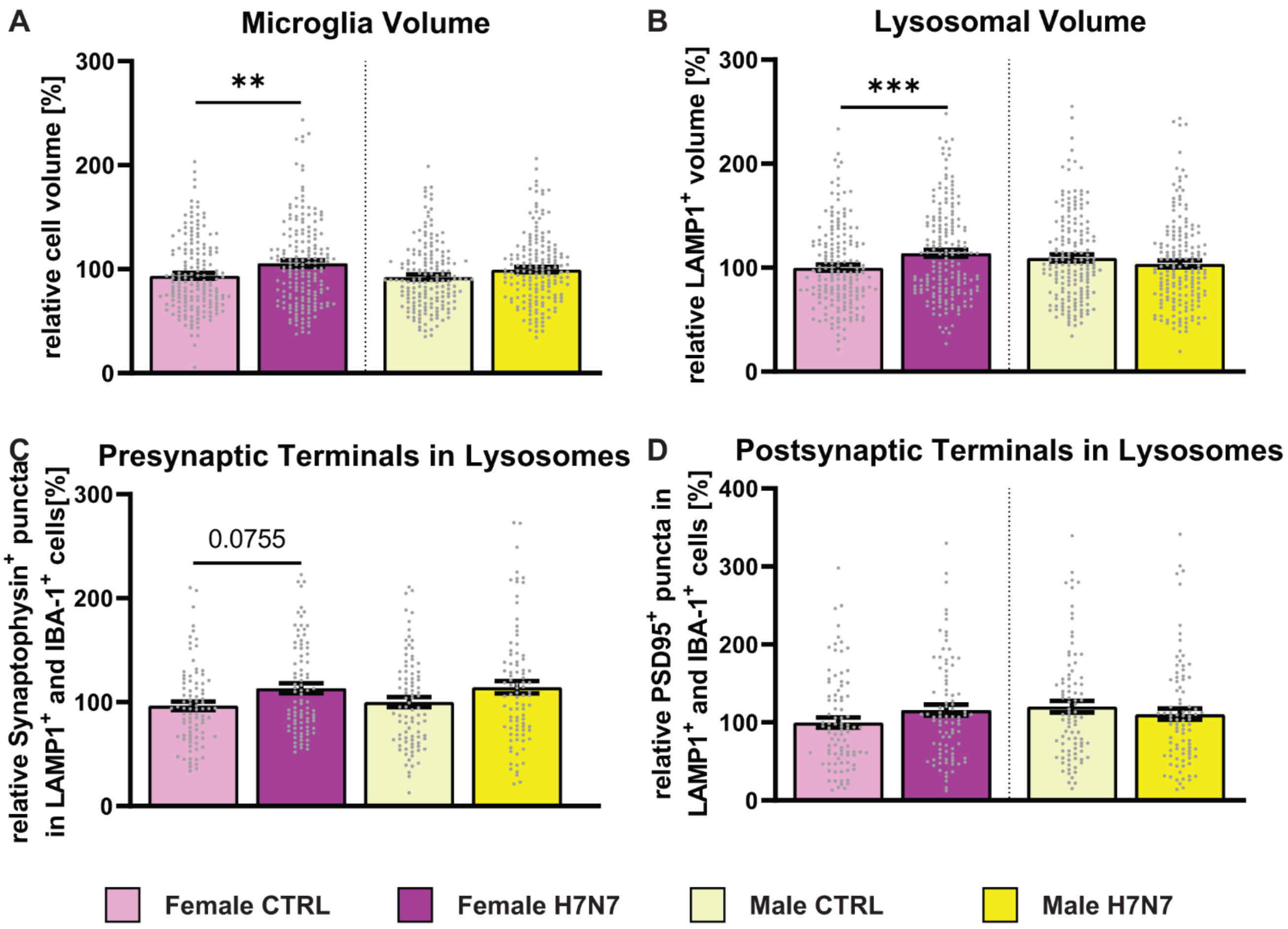

Supplementary Figure 6. Engulfment of excitatory synaptic terminals 6 h after infection with neurotropic IAV H7N7. **(A)** Increased microglia cell volume in female-derived cultures was seen at 6 hpi, whereby no volumetric changes in male-derived cultures occurred. **(B)** An increase in the lysosomal volume in female-derived cultures was observed as early as 6 hpi indicating an increased phagocytic activity. Number of experiments, N = 6, n = 30 cells per group and cell preparation round. **(C)** A trend indicating a starting increase in the engulfment of presynaptic terminals was observed in female-derived cultures 6 hpi. **(D)** No changes in the engulfment of postsynaptic terminals could be observed at 6 hpi. Number of experiments, N = 3, n = 30 cells per group and cell preparation round. Data are presented as mean ± SEM and were analyzed with an ordinary two-way ANOVA with post hoc Tukey test. **p < 0.01 and ***p < 0.001.

Table S1 – Statistical Information: Mean level.

Figure	Mean level of …			
**Fig. 1B**	Female 6 hpi	Female 24 hpi	Male 6 hpi	Male 24 hpi
Total Infection Rate with microglia	4.877 ± 0.3547	12.56 ± 0.8319	7.976 ± 0.7515	12.45 ± 0.9432
**Fig. 1C**	Female 6 hpi	Female 24 hpi	Male 6 hpi	Male 24 hpi
Total Infection Rate without microglia	1.559 ± 0.1781	9.874 ± 0.5760	0.9981 ± 0.1324	8.130 ± 0.5442
**Fig. 3D**	Female CTRL Amoeboid 6 h	Female CTRL Amoeboid 24 h	Female H7N7 Amoeboid 6 h	Female H7N7 Amoeboid 24 h
Microglia Morphology	31.93 ± 2.002	36.47 ± 2.551	35.20 ± 3.308	26.83 ± 4.279
	Male CTRL Amoeboid 6 h	Male CTRL Amoeboid 24 h	Male H7N7 Amoeboid 6 h	Male H7N7 Amoeboid 24 h
	29.48 ± 2.049	26.72 ± 2.424	36.93 ± 3.287	38.66 ± 4.121
	Female CTRL Intermediate 6 h	Female CTRL Intermediate 24 h	Female H7N7 Intermediate 6 h	Female H7N7 Intermediate 24 h
	51.90 ± 1.574	46.50 ± 2.878	38.24 ± 2.822	39.29 ± 4.802
	Male CTRL Intermediate 6 h	Male CTRL Intermediate 24 h	Male H7N7 Intermediate 6 h	Male H7N7 Intermediate 24 h
	43.41 ± 2.710	53.15 ± 2.780	29.43 ± 2.712	27.84 ± 3.312
	Female CTRL Ramified 6 h	Female CTRL Ramified 24 h	Female H7N7 Ramified 6 h	Female H7N7 Ramified 24 h
	16.18 ± 2.024	13.70 ± 1.835	23.23 ± 2.659	30.55 ± 4.975
	Male CTRL Ramified 6 h	Male CTRL Ramified 24 h	Male H7N7 Ramified 6 h	Male H7N7 Ramified 24 h
	27.11 ± 2.704	18.64 ± 1.491	26.98 ± 2.897	33.50 ± 4.326
**Supplementary Fig. 4B**	Female CTRL with microglia	Female H7N7 with microglia	Male CTRL with microglia	Male H7N7 with microglia
Spine Density 6 h	0.6910 ± 0.01577	0.7479 ± 0.01620	0.7209 ± 0.01767	0.7259 ± 0.01895
	Female CTRL without microglia	Female H7N7 without microglia	Male CTRL without microglia	Male H7N7 without microglia
	0.7173 ± 0.02008	0.7697 ± 0.01843	0.7004 ± 0.01662	0.7745 ± 0.02022
**Fig. 5B**	Female CTRL with microglia	Female H7N7 with microglia	Male CTRL with microglia	Male H7N7 with microglia
Spine Density 24 h	0.6780 ± 0.01807	0.7407 ± 0.01955	0.6553 ± 0.01811	0.6842 ± 0.02113
	Female CTRL without microglia	Female H7N7 without microglia	Male CTRL without microglia	Male H7N7 without microglia
	0.7257 ± 0.02251	0.8063 ± 0.02269	0.7509 ± 0.02268	0.8017 ± 0.02520
**Supplementary Fig. 4C**	Female CTRL with microglia	Female H7N7 with microglia	Male CTRL with microglia	Male H7N7 with microglia
Functional Synapse 6 h	4.698 ± 0.4871	6.732 ± 0.6567	4.185 ± 0.3980	4.789 ± 0.5130
	Female CTRL without microglia	Female H7N7 without microglia	Male CTRL without microglia	Male H7N7 without microglia
	6.092 ± 0.5885	3.314 ± 0.3040	4.948 ± 0.4855	3.407 ± 0.3487
**Fig. 5C**	Female CTRL with microglia	Female H7N7 with microglia	Male CTRL with microglia	Male H7N7 with microglia
Functional Synapse 24h	4.698 ± 0.4871	7.397 ± 0.8007	5.829 ± 0.7904	4.923 ± 0.4976
	Female CTRL without microglia	Female H7N7 without microglia	Male CTRL without microglia	Male H7N7 without microglia
	6.092 ± 0.5885	6.648 ± 0.5682	5.712 ± 0.4660	6.794 ± 0.6957

Table S2 – Statistical Information: Two-way ANOVA.

**Figure**	**Two-way ANOVA**		
**Fig. 2A**	$Sex_{+microglia}$	$Infection_{+microglia}$	Sex x $Infection_{+microglia}$
IFN-β 6 hpi	F (1, 12) = 0.1029, P=0.7538	F (1, 12) = 12.01, P=0.0047	F (1, 12) = 0.0002929, P=0.9866
	$Sex_{-microglia}$	$Infection_{-microglia}$	Sex x $Infection_{-microglia}$
	F (1, 12) = 2.804, P=0.1199	F (1, 12) = 36.69, P<0.0001	F (1, 12) = 1.791, P=0.2056
**Fig. 2B**	$Sex_{+microglia}$	$Infection_{+microglia}$	Sex x $Infection_{+microglia}$
IFN-β 24 hpi	F (1, 12) = 5.024, P=0.0447	F (1, 12) = 2.292, P=0.1559	F (1, 12) = 5.589, P=0.0358
	$Sex_{-microglia}$	$Infection_{-microglia}$	Sex x $Infection_{-microglia}$
	F (1, 12) = 6.457, P=0.0259	F (1, 12) = 115.6, P<0.0001	F (1, 12) = 6.457, P=0.0259
**Fig 2C**	$Sex_{+microglia}$	$Infection_{+microglia}$	Sex x $Infection_{+microglia}$
IL-6 6 hpi	F (1, 15) = 0.2733, P=0.6087	F (1, 15) = 8.769, P=0.0097	F (1, 15) = 0.4062, P=0.5335
	$Sex_{-microglia}$	$Infection_{-microglia}$	Sex x $Infection_{-microglia}$
	F (1, 16) = 1.080, P=0.3141	F (1, 16) = 0.1455, P=0.7079	F (1, 16) = 0.8643, P=0.3664
**Fig 2D**	$Sex_{+microglia}$	$Infection_{+microglia}$	Sex x $Infection_{+microglia}$
IL-6 24 hpi	F (1, 16) = 0.1917, P=0.6674	F (1, 16) = 16.84, P=0.0008	F (1, 16) = 0.01151, P=0.9159
**Fig 2E**	$Sex_{+microglia}$	$Infection_{+microglia}$	Sex x $Infection_{+microglia}$
TNF-α 6 hpi	F (1, 24) = 0.3697, P=0.5489	F (1, 24) = 17.28, P=0.0004	F (1, 24) = 0.07263 , P=0.7899
	$Sex_{-microglia}$	$Infection_{-microglia}$	Sex x $Infection_{-microglia}$
	F (1, 23) = 0.8689, P=0.3609	F (1, 23) = 2.647, P=0.1174	F (1, 23) = 1.688, P=0.2068
**Fig. 2F**	$Sex_{+microglia}$	$Infection_{+microglia}$	Sex x $Infection_{+microglia}$

TNF-α 24 hpi	F (1, 16) = 0.5244, P=0.4794	F (1, 16) = 55.48, P<0.0001	F (1, 16) = 0.3467, P=0.5642
	$Sex_{-microglia}$	$Infection_{-microglia}$	Sex x $Infection_{-microglia}$
	F (1, 24) = 0.9772, P=0.3328	F (1, 24) = 2.025, P=0.1676	F (1, 24) = 0.9772, P=0.3328
**Fig 2G**	$Sex_{+microglia}$	$Infection_{+microglia}$	Sex x $Infection_{+microglia}$
CCL2 6 hpi	F (1, 21) = 7.811, P=0.0109	F (1, 21) = 90.25, P<0.0001	F (1, 21) = 20.88, P=0.0002
	$Sex_{-microglia}$	$Infection_{-microglia}$	Sex x $Infection_{-microglia}$
	F (1, 20) = 0.004541, P=0.9469	F (1, 20) = 0.001706, P=0.9675	F (1, 20) = 0.001133, P=0.9735
**Fig 2H**	$Sex_{+microglia}$	$Infection_{+microglia}$	Sex x $Infection_{+microglia}$
CCL2 24 hpi	F (1, 22) = 0.2040, P=0.6560	F (1, 22) = 17.39, P=0.0004	F (1, 22) = 0.01549, P=0.9021
	$Sex_{-microglia}$	$Infection_{-microglia}$	Sex x $Infection_{-microglia}$
	F (1, 9) = 0.3499, P=0.5687	F (1, 9) = 1.200, P=0.3018	F (1, 9) = 0.1301, P=0.7266
**Fig. 2I**	$Sex_{+microglia}$	$Infection_{+microglia}$	Sex x $Infection_{+microglia}$
CCL5 6 hpi	F (1, 12) = 3.505, P=0.0857	F (1, 12) = 110.1, P<0.0001	F (1, 12) = 6.947, P=0.0217
	$Sex_{-microglia}$	$Infection_{-microglia}$	Sex x $Infection_{-microglia}$
	F (1, 11) = 2.246, P=0.1621	F (1, 11) = 2.246, P=0.1621	F (1, 11) = 2.246, P=0.1621
**Fig. 2J**	$Sex_{+microglia}$	$Infection_{+microglia}$	Sex x $Infection_{+microglia}$
CCL5 24 hpi	F (1, 12) = 8.477, P=0.0130	F (1, 12) = 332.9, P<0.0001	F (1, 12) = 12.57, P=0.0040
**Fig. 4B**	$Sex_{amoeboid}$	$Infection_{amoeboid}$	Sex x $Infection_{amoeboid}$
IBA1 6 hpi	F (1, 112) = 0.07276, P=0.7879	F (1, 112) = 0.02607, P=0.8720	F (1, 112) = 0.5665, P=0.4532
	$Sex_{ramified}$	$Infection_{ramified}$	Sex x $Infection_{ramified}$
	F (1, 107) = 1.784, P=0.1846	F (1, 107) = 0.5969, P=0.4414	F (1, 107) = 0.1603, P=0.6897
**Fig. 4C**	$Sex_{amoeboid}$	$Infection_{amoeboid}$	Sex x $Infection_{amoeboid}$

IBA1 24 hpi	F (1, 100) = 1.335, P=0.2507	F (1, 100) = 1.708, P=0.1943	F (1, 100) = 13.34, P=0.0004
	$Sex_{ramified}$	$Infection_{ramified}$	Sex x $Infection_{ramified}$
	F (1, 86) = 8.615, P=0.0043	F (1, 86) = 0.006407, P=0.9364	F (1, 86) = 16.24, P=0.0001
**Fig. 4D**	$Sex_{amoeboid}$	$Infection_{amoeboid}$	Sex x $Infection_{amoeboid}$
CD68 6 hpi	F (1, 113) = 0.5926, P=0.4430	F (1, 113) = 1.059, P=0.3057	F (1, 113) = 0.02659, P=0.8708
	$Sex_{ramified}$	$Infection_{ramified}$	Sex x $Infection_{ramified}$
	F (1, 103) = 2.139, P=0.1466	F (1, 103) = 0.002452, P=0.9606	F (1, 103) = 0.04393, P=0.8344
**Fig. 4E**	$Sex_{amoeboid}$	$Infection_{amoeboid}$	Sex x $Infection_{amoeboid}$
CD68 24 hpi	F (1, 99) = 2.581, P=0.1113	F (1, 99) = 20.23, P<0.0001	F (1, 99) = 0.8234, P=0.3664
	$Sex_{ramified}$	$Infection_{ramified}$	Sex x $Infection_{ramified}$
	F (1, 87) = 6.647, P=0.0116	F (1, 87) = 3.220, P=0.0762	F (1, 87) = 4.493, P=0.0369
**Fig. 4F**	Sex	Infection	Sex x Infection
Microglia cell volume 24 hpi	F (1, 716) = 0.5384, P=0.4633	F (1, 716) = 8.967, P=0.0028	F (1, 716) = 0.04751, P=0.8275
**Fig. 4G**	Sex	Infection	Sex x Infection
Lysosomal volume 24 hpi	F (1, 705) = 0.2571, P=0.6123	F (1, 705) = 49.71, P<0.0001	F (1, 705) = 0.7815, P=0.3770
